# Spacial Score—A Comprehensive Topological Indicator for Small-Molecule Complexity

**DOI:** 10.1021/acs.jmedchem.3c00689

**Published:** 2023-08-31

**Authors:** Adrian Krzyzanowski, Axel Pahl, Michael Grigalunas, Herbert Waldmann

**Affiliations:** † Department of Chemical Biology, Max Planck Institute of Molecular Physiology, Otto-Hahn-Straße 11, 44227 Dortmund, Germany; ‡ Faculty of Chemistry, Chemical Biology Technical University Dortmund, Otto-Hahn-Straße 6, 44221 Dortmund, Germany; § Compound Management and Screening Center, Max Planck Institute of Molecular Physiology, Otto-Hahn-Straße 11, 44227 Dortmund, Germany

## Abstract

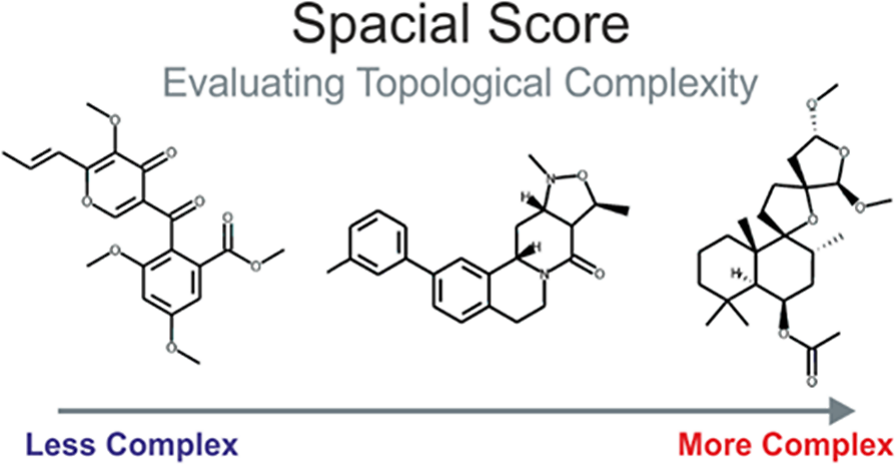

The fraction of sp^3^-hybridized carbons (*F*_sp^3^_) and the fraction of stereogenic carbons (*F*_Cstereo_) are two widely employed scores of molecular complexity with strong links to biologically relevant features. However, they do not comprehensively express molecular topology, and they often do not match the chemical intuition of complexity. We propose the spacial score (SPS) as an empirical scoring system that builds upon the principle underlying *F*_sp^3^_ and *F*_Cstereo_ and expresses the spacial complexity of a compound in a uniform manner on a highly granular scale. The size-normalized SPS (nSPS) can differentiate distributions of natural products and synthetic compounds and is applicable in the analysis of biological activity data. Analysis of the ChEMBL database revealed general trends of increasing selectivity and potency with increasing nSPS. SPS can also be used advantageously in planning and analysis of synthesis programs for direct comparison of chemical transformations and intermediates in reaction sequences.

## Introduction

For the objective comparison of small structures, several indicators and descriptors have been devised which aim to express various characteristics of compounds, such as physicochemical properties, shape, topology, and total molecular complexity. Such indicators may be invaluable in synthesis planning and analysis of compound libraries.^1^ Complexity descriptors attempt to translate intuitive qualitative features describing a property into quantitative measures, and both individual intuitive features and complexity descriptors have their own biases. Additionally, a relationship between molecular complexity and biological activity was initially indicated by the theoretical model of Hann et al.^2^ and the experiments performed by Kuntz et al.,^3^ highlighting the importance of complexity indicators in medicinal chemistry. Thus, complexity metrics that not only match chemical intuition but also correlate to biological outcomes, such as target selectivity, are of particular value to the drug discovery community.

Two informative and powerful indicators of molecular shape complexity are the fraction of sp^3^-hybridized carbons (*F*_sp^3^_) and the fraction of stereogenic carbons (*F*_Cstereo_). The *F*_sp^3^_ index, as defined by Lovering et al.,^4^ is a size-normalized measure where the number of sp^3^ carbons is divided by the total number of carbons in a given organic molecule:
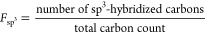
1Lovering et al. suggested that a higher fraction of saturation in molecules results in the coverage of more diverse structural space and improved potential to identify compounds with spacial complementarity matching the pockets of target proteins. An increase in *F*_sp^3^_ can result in a higher architectural complexity of molecules without an increase in the number of heavy atoms by allowing access to a larger number of isomers and broader conformational diversity. It was proposed that a potential improvement in the three-dimensionality of molecules caused by an increase in saturation of the skeleton could result in greater selectivity and a decrease in off-target interactions. Thus, Lovering et al. showed a clear trend where compounds with a higher *F*_sp^3^_ are more likely to succeed in advancing from the discovery stage to clinical trials and ultimately to yield marketed drugs.^4^ Subsequently, Lovering demonstrated that the promiscuity of compounds in different assays decreased as a function of *F*_sp^3^_.^5^ This finding was later confirmed, and a trend of improvement in protein binding selectivity and frequency for compounds with higher proportions of sp^3^ carbons was recorded.^6−8^ As increasing specificity and selectivity typically reduce the likelihood of toxicity due to the minimization of off-target effects,^9^ an increase in *F*_sp^3^_ of synthesized compounds could also result in a general reduction of toxicity.^5^

*F*_Cstereo_ is a size-independent score, defined as the number of stereogenic carbons divided by the total number of all carbons (eq 2). In the context of the *F*_Cstereo_ indicator, the stereogenic carbons are assumed to be carbons which are connected to four different groups:
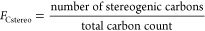
2Clemons et al.^6^ found that protein-binding frequency and selectivity increased for small molecules with intermediate proportions of stereogenic carbons, in comparison to compounds with very low and high stereogenic content, indicating *F*_Cstereo_ as an important score for consideration in designing screening libraries. These selectivity results are also supported by other reports noting an improvement of selectivity with the increase in stereochemical complexity.^7,8^

Various 3D descriptors correlate well with compound selectivity, observed solubility, and clinical success.^7^ However, a major drawback of calculating 3D descriptors is the necessity for accurate prediction of molecular conformations, which can pose a significant computational challenge and cost for the analysis of larger compound libraries.

A number of elaborate scoring systems has also been devised to express topological and total molecular complexity.^10−23^ However, arguably, the most popular general scoring system for molecular complexity has been introduced by Bertz,^10^ considering both the topological features of a molecular skeleton through the *C*(η) score calculated for subgraphs of a molecular graph and its elemental content via the *C*(*E*) component accounting for the presence of heteroatoms. The Bertz total molecular complexity index *C*_T_ is then simply calculated through summation of the topological and the elemental diversity scores, where *C*_T_ = *C*(η) + *C*(*E*). The Bertz complexity index is used as a molecular complexity measure for compound evaluation by Pubchem, a popular public repository of chemical information.^24,25^ Another exemplary complexity scoring system based on connectivity of subgraphs was proposed by Bonchev,^11^ resulting in two complexity indices, TC and TC1.

Complexity measures based on the analysis of substructures include, for instance, the empirical and intuitive system devised by Whitlock,^16^ which takes into consideration the number of alicyclic rings, non-aromatic unsaturations, heteroatoms, and stereogenic centers. Here the complexity score *S* is a sum of the appropriately weighted numbers of given components with no regard for the skeletal connectivity. A very similar approach and modification of Whitlock’s complexity system was presented by Barone and Chanon,^17^ who in their scoring index additionally account for the number of substituents and ring sizes. Complexity metrics based on other features include the score of Allu and Oprea^18^ relying on atomic electronegativities and bond parameters or the metric of von Korff and Sander^19^ calculating a fractal dimension of a molecule. Complexity focusing on the molecular shape can also be described by the Kappa shape indices through a comparison of the analyzed molecule to the extreme shapes for a structure with the same number of atoms.^20^ The Kappa indices are calculated with respect to a fully linear and fully connected structure, as well as to a star-shaped scaffold. The disadvantage of the Kappa scoring system is the necessity for comparison of different indices, which can be inconvenient for the analysis of larger molecular libraries.

Recently, Böttcher^21^ tried to overcome many shortcomings of the existing scoring systems and devised a new approach based on substructure analysis, intended for expression of the total molecular complexity. The Böttcher score *C*_m_ is inspired by the Shannon entropy and defines molecular complexity in an additive manner by the consideration of atomic connectivity, the number of valence electrons for atoms, heteroatom diversity, stereogenicity, and molecular symmetry. It has generated significant interest, resulting in a rapid increase in its popularity since its introduction, notably by the organic synthesis community.^26−32^ Considering the general interest in this scoring system and apparent improvement over the alternative complexity measures, we explored whether the Böttcher complexity score may also serve as a descriptor that could correlate compound complexity with biological activity and selectivity. In this case, it might be employed to rationalize trends observed in the biological analysis of our in-house compound libraries. However, initial analysis using total and size-normalized scores indicated that, although the Böttcher complexity index is intuitive and very applicable in a chemical context, there seems to be no or at best only very limited correlation to biological compound activity and selectivity (see the Supporting Information). In light of the recent report by Méndez-Lucio and Medina-Franco^8^ investigating the relationship between different complexity measures and selectivity, we concluded that scores such as the Bertz molecular complexity also do not seem to be broadly applicable in a biological context.

Considering the already proven correlation between *F*_sp^3^_ and *F*_Cstereo_ indicators and biologically relevant characteristics of compounds, we now propose a new empirical scoring system termed the spacial score (SPS). [*Note:* The term “spacial” is the British English spelling of the word “spatial” in American English. Both words originate from the Latin word “spatium”, which translates to “space”.] The SPS builds and improves upon the individual *F*_sp^3^_ and *F*_Cstereo_ indicators by uniformly expressing spacial complexity on a highly granular scale for convenient ranking of and comparison between molecules. Furthermore, the SPS is an easy-to-calculate index that does not require information about the 3D conformation of a molecule and, when normalized, correlates to biologically relevant properties, including selectivity and potency.

## Results and Discussion

The SPS is based on four molecular parameters that we consider significant, i.e., an atom hybridization term (*h*), a stereoisomeric term (*s*), a non-aromatic ring term (*r*), and the number of heavy-atom neighbors (*n*). By design, the score does not account for heteroatom diversity, as it is not intended to capture the total molecular complexity. The terms are calculated for each heavy atom in a molecule and are then summed across the whole structure according to eq 3, where the symbol *i* denotes the heavy-atom index in a molecule. The total score value is intended to increase with an increase in the relative complexity of the skeletal arrangements.
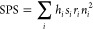
3The *h* term accounts for the conformational degrees of freedom. *h* equals 3, 2, and 1 for sp^3^-, sp^2^-, and sp-hybridized atoms, respectively, and *h* is assumed to be 4 for any other type of hybridization, such as sp^*x*^d^*y*^ and sd^*x*^ in transition metals. Thus, the term *h* is directly related to *F*_sp^3^_, for which typically a saturated compound with a high content of sp^3^ carbons receives a higher score than a similar unsaturated structure. SPS also accounts for the stereogenicity of an atom, where (pseudo)stereogenic tetrahedral carbons and atoms involved in a double bond with possible *E* and *Z* isomers are assigned *s* = 2, and otherwise the term is equal to 1. The stereoisomeric term *s* is thus strongly related to *F*_Cstereo_. The value of the ring term *r* is 2 for all atoms being a part of a non-aromatic ring system. Atoms which are part of an aromatic ring or linear structure are scored with *r* = 1. The different score for non-aromatic compounds reflects that corresponding elements typically have more spatially intriguing features and conformations. Finally, the score accounts for the number of heavy-atom neighbors of the atom under consideration. For example, an atom with bonds to two heavy atoms has *n* = 2, and an atom with connections to three heavy atoms is assigned *n* = 3. The branching of the molecular skeleton is accounted for by squaring the *n* term.

Table 1 shows the scoring system for atoms of a representative small molecule. The possible value range of SPS starts at 0 and has no upper limit. However, in order for the SPS to be directly comparable among diverse structures, molecular size needs to be taken into consideration. The SPS can be easily and reliably normalized with respect to size by dividing the total score by the total number of heavy atoms (*a*) in a given molecule, and the resulting normalized score is referred to as nSPS (eq 4). The SPS and nSPS computations were implemented as a Python script based on the RDKit software,^33^ calculating the scores using simplified molecular-input line-entry system (SMILES) as input.^34^
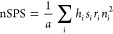
4The SPS and nSPS were compared to the frequently used complexity indices *F*_sp^3^_, *F*_Cstereo_, Böttcher complexity score *C*_m_, Bertz total molecular complexity *C*_T_, and Whitlock score *S*. The results for a set of representative compounds are shown in Table 2. *F*_sp^3^_ and *F*_Cstereo_ are straightforward indicators and therefore can often return the lowest possible score of 0, even for molecules with considerable topological complexity. *F*_sp^3^_ can also often yield the highest score of 1 for simple structures (Figure S1). Additionally, a significant portion of small molecules does not have any stereogenic carbons, resulting in *F*_Cstereo_ scores of 0. Four different reference sets were curated and used for analysis, i.e., experimental and approved drugs from the DrugBank (DrugBank),^35^ the Enamine Advanced Screening Collection (Enamine, representing a commercial screening collection optimized for Lipinski’s rule of five),^36^ natural products extracted from the ChEMBL database (ChEMBL NPs, representing bioactive natural products),^37^ and dark chemical matter (DCM, representing compounds that do not show activity in >100 biological and biochemical assays).^38^ The results show that 56% of all structures have 1 or more stereogenic carbons and, consequently, 44% of the molecules have an *F*_Cstereo_ score of 0 (Figure S2). Thus, *F*_Cstereo_ is not an ideal indicator for comparisons and ranking among a significant number of compounds in such libraries. Unsurprisingly, *F*_sp^3^_ and *F*_Cstereo_ indicators do not directly encapsulate many topological features, such as the extent of skeletal branching or the presence of rings (see Table 2), and thus, they are not comprehensive measures of complexity.

**Table 1:**
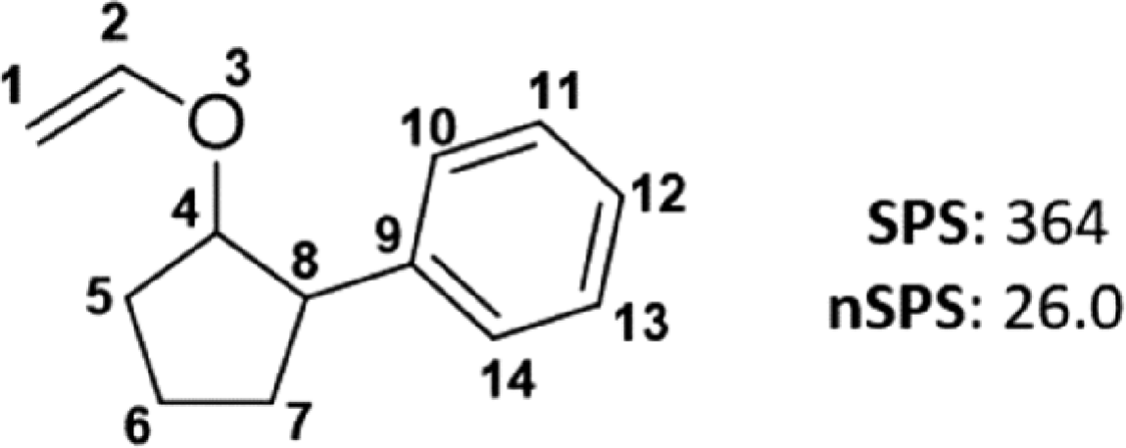

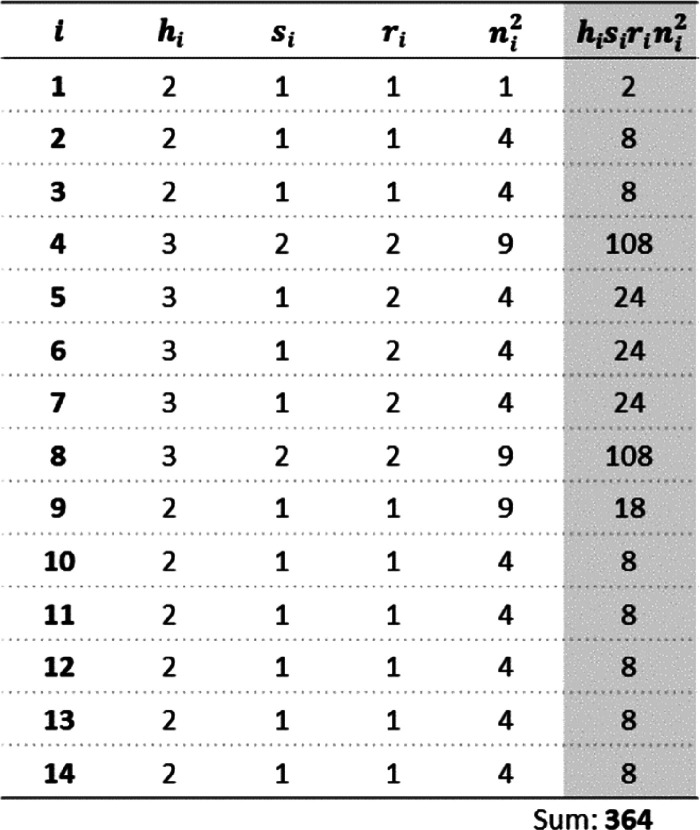
Illustration of SPS Partial Scores for Every Atomic Index in the Depicted Small Molecule^a^

a The total SPS calculated for the compound is 364, and the nSPS is equal to 26.0. Abbreviations: *i*, atom index; *h*, hybridization term; *s*, stereoisomeric term; *r*, non-aromatic ring term; *n*, heavy-atom neighbors.

**Table 2:**
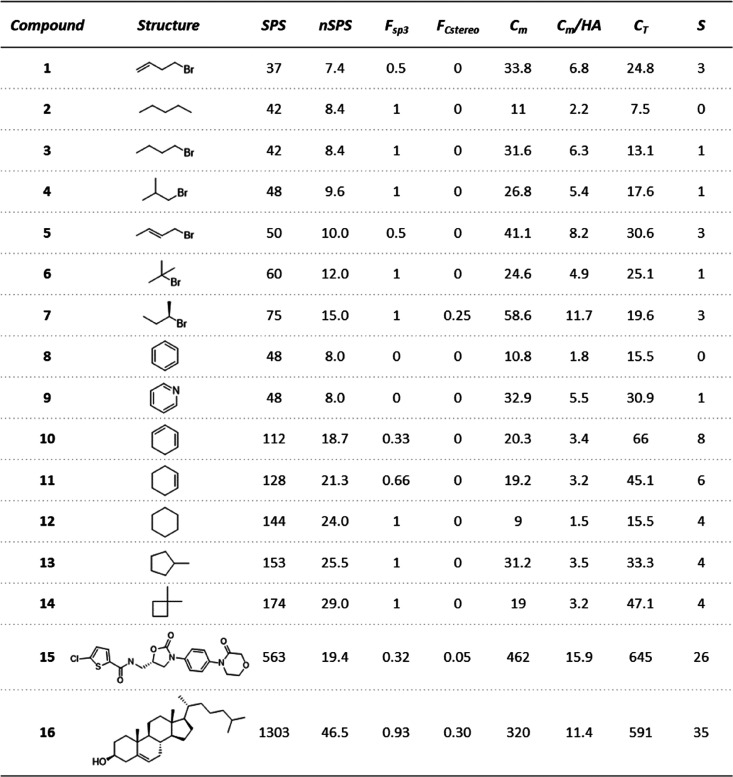
Comparison of SPS and nSPS with Scores Obtained with Common Topological and Total Complexity Indicators for Exemplary Small Organic Molecules^a^

a Abbreviations: *C*_m_, Böttcher complexity score; *C*_T_, Bertz total molecular complexity; HA, heavy atoms; *S*, Whitlock score.

In contrast, complexity scores like *S*, *C*_T_, or *C*_m_ are intended to comprehensively express the total molecular complexity. The Whitlock measure *S* does not differentiate well between compounds containing a smaller number of features and is indifferent toward many important topological features, including branching and ring sizes. On the other hand, the *C*_T_ index expresses a number of topological nuances, but this scoring system is limited, for instance, by not taking chirality into account. Additionally, a very strong correlation between log_10_ of *C*_T_ scores and molecular weight has been reported (Spearman’s rank correlation coefficient equal to 0.98).^39^*C*_m_ appears to score compounds in a more intuitive manner, easily distinguishing between linear, branched, saturated, and unsaturated compounds with consideration for symmetry and elemental diversity. This complexity index, however, puts an emphasis on the presence of heteroatoms, as can be exemplified by comparison of *n*-hexane (size-normalized *C*_m_ by accounting for number of heavy atoms: *C*_m_/HA = 2.2) with *n*-amyl bromide (*C*_m_/HA = 6.3) or between more elaborate compounds such as rivaroxaban (*C*_m_/HA = 15.9; compound **15** in Table 2) and cholesterol (*C*_m_/HA = 11.4; compound **16** in Table 2). The nSPS has also been compared to the interpretation of the Allu and Oprea scoring system^18^ by Voršilák and Svozil, relying on atomic electronegativities and bond parameters (Figure S3).^,40^ The analysis showed no considerable correlation between the two scoring methods.

**Figure 1:**
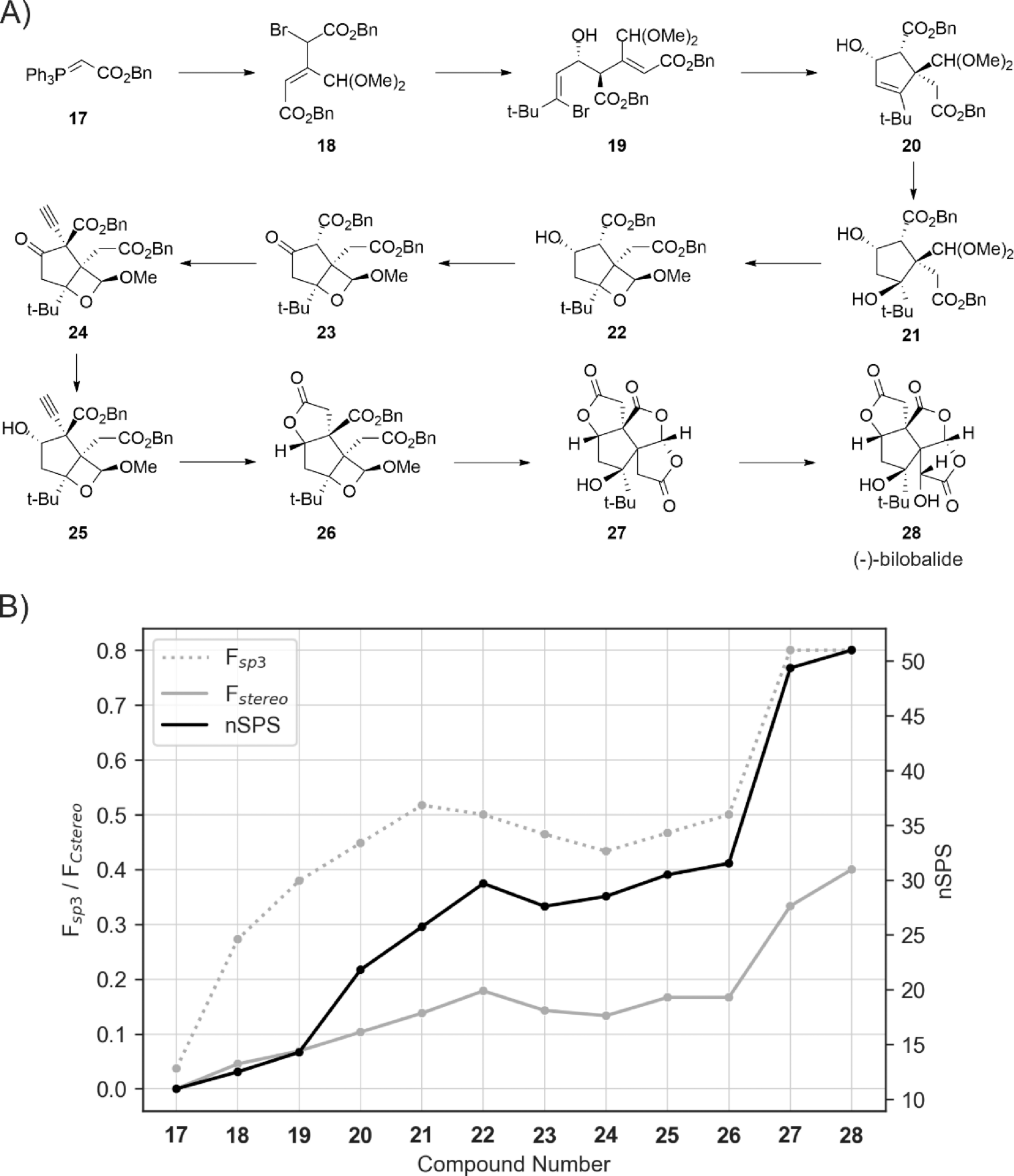
**A)** Overview of the total synthesis of (−)-bilobalide performed by Demoret et al.^26^**B)***F*_sp^3^_, *F*_Cstereo_, and nSPS calculated for the structures in the total synthesis of (−)-bilobalide.

SPS and nSPS are indifferent to the elemental constitution of molecules (compare compounds **2** and **3** in Table 2) and penalize unsaturation (compare **1** and **3**), unless it contributes to the isomeric diversity (compound **5**). Branched and non-aromatic cyclic molecules are awarded higher scores than linear structures (e.g., compare compounds **3** and **4**, as well as **2** and **12**), and stereogenic atoms are stressed (compound **7**). Comparison of the nSPS of cholesterol (46.5) to the nSPS of rivaroxaban (19.4; Table 2), as well as various molecules at different nSPS ranges (Figures S4 and S5), matches the general intuition concerning the intricacy of spatial arrangements in these molecules. We also calculated and compared *F*_sp^3^_, *F*_Cstereo_, and nSPS for each of the synthesized compounds in the total synthesis of (−)-bilobalide by Demoret et al.,^26^ further substantiating our conclusion regarding the intuitive nature of the new scoring system and combined improvement over *F*_sp^3^_ and *F*_Cstereo_ as individual descriptors (Figure 1).

**Figure 2:**
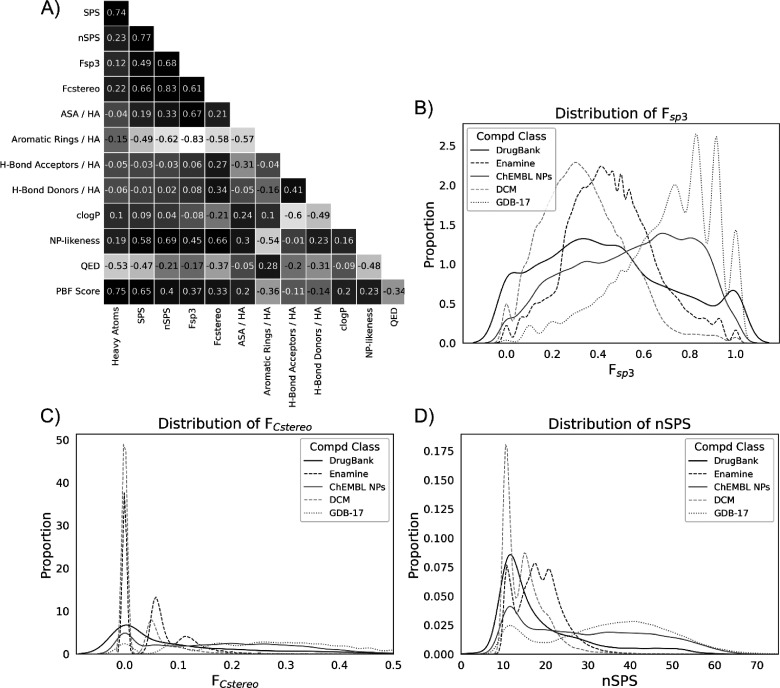
**A)** Correlation heatmap constructed based on the analysis of 12 000 representative organic molecules selected in equal proportions from DrugBank, Enamine, ChEMBL NP, and DCM data sets. The values shown are calculated Pearson correlation coefficients between descriptors. **B)** Kernel distribution estimation plot (KDE) depicting the distribution of *F*_sp^3^_ for different data sets. **C)** KDE for *F*_Cstereo_ for different compound classes. **D)** KDE plot of calculated nSPS values for different data sets. Abbreviations: ASA, approximate surface area; DCM dark chemical matter; HA, heavy atoms; NPs, natural products; PBF, plane of best fit; QED, quantitative estimate of drug-likeness.

Assessment of Pearson correlation coefficients of nSPS with basic molecular descriptors for a collection of representative molecules from the DrugBank, Enamine, ChEMBL NP, and DCM reference sets revealed that, as designed, the score is highly correlated with *F*_sp^3^_ (0.68) and *F*_Cstereo_ (0.83) and negatively correlated with the size-normalized number of aromatic rings (−0.62; Figure 2A). Further analysis showed that, in order to reach high nSPS, compounds need to have high *F*_sp^3^_ scores; however, high *F*_sp^3^_ scores do not necessarily translate to large nSPS values (Figure S6A). Similarly, not all compounds with high *F*_Cstereo_ are complex, as determined based on the corresponding nSPS, and molecules with *F*_Cstereo_ = 0 can also have elaborate topology, as discussed above (Figure S6B). No correlation with the size-normalized approximate surface area (ASA),^41^ number of hydrogen bond donors and acceptors, estimated clogP values,^42^ and quantitative estimate of drug-likeness (QED) was observed.^43^ However, we noted a significant correlation (0.69) with natural product-likeness (NP-likeness),^44^ as well as a moderate degree of correlation (0.4) with the plane of best fit (PBF) score (Figure 2).^45^ The relationship with the PBF score, although not significantly stronger than what is observed for *F*_sp^3^_ and *F*_Cstereo_, is interesting, as calculations of PBF are normally computationally demanding due to the necessity to accurately predict the representative 3D conformations of the investigated molecules.

*F*_sp^3^_, *F*_Cstereo_, and nSPS were calculated for comparison for all compounds from the different data sets (Figure 2B–D). Interestingly, we observed that a high proportion of the DCM compound collection shows relatively low nSPS, with a median score of 14.3. DrugBank gave a relatively high overlap with DCM, with a median score of 14.4; however, its distribution also showed a very long tail, with the maximum nSPS values reaching 66.4. Compounds from the Enamine collection had a higher median nSPS than DCM and DrugBank molecules that was equal to 18.2. There is a general chemical intuition that NPs produced via biosynthetic cascades are typically more complex than synthetic molecules produced via chemical reactions.^46−51^ In line with this intuition, ChEMBL NPs have a very high nSPS median value (28.0) and a relatively broad distribution (maximum nSPS of 76.0) relative to the other reference sets. The wide range of nSPS values for NPs expresses their rich structural diversity, which includes such simple molecules like salicylic and cinnamic acids but also extends to more intricate compounds such as morphine or strychnine. We also analyzed a representative subset of the GDB-17 enumerated compound library, containing molecules with up to 17 atoms of C, N, O, S, and halogens. The GDB-17 set is known to be rich in non-aromatic cyclic structures, compounds with a high proportion of quaternary centers and stereoisomers. The shapes of the compounds from this set also have been shown to significantly populate the third dimension.^52^ The GDB-17 subset has a similar distribution of the nSPS values as the analyzed NPs. The median score is 36.2 units and confirms the very high complexity of the set, where the most complex structures are found to have nSPS of over 100. Despite its more structurally holistic approach, nSPS still conserves the characteristic distinction between the distributions of synthetic compounds and NPs as it is seen for *F*_Cstereo_. An analogous analysis employing size-normalized *C*_m_ scores could not differentiate the reference compound sets (Figure S11). The relatively high *F*_Cstereo_ and *F*_sp^3^_ values of NPs may explain the significant correlation of nSPS to the NP-likeness score.

In order to determine if the nSPS scores are related to the general trends in compound activity and selectivity, and how those trends relate to *F*_sp^3^_ and *F*_Cstereo_, we analyzed the ChEMBL database, selecting only for molecules tested in assays with the highest confidence score. Retention of the assay outcomes with the highest confidence score assured that the used results are accurate and the target assignments represent the actual targets. Compound activity was assessed based on the averaged available pChEMBL values for a given molecule. The pChEMBL value is a negative log of concentration–response activity such as IC_50_, EC_50_, *K*_i_, or *K*_d_. The analyzed structures were classified as compounds with low (average pChEMBL ≤ 5.3), medium (5.3 < average pChEMBL < 6.5), or high activity (average pChEMBL ≥ 6.5).

Compounds were further assigned into three complexity levels, depending on the considered scoring system. Based on *F*_sp^3^_ index, molecules were classified using score thresholds of 0.3 and 0.6, and based on *F*_Cstereo_, using thresholds of 0.0 and 0.25. The selected *F*_Cstereo_ classification values also matched the categorization values used previously by Clemons et al.^6^ Compounds were assumed to have low nSPS when the score was below 11.2 (25th percentile of the analyzed ChEMBL compounds) or high nSPS with the value above 17.7 (75th percentile).

**Figure 3:**
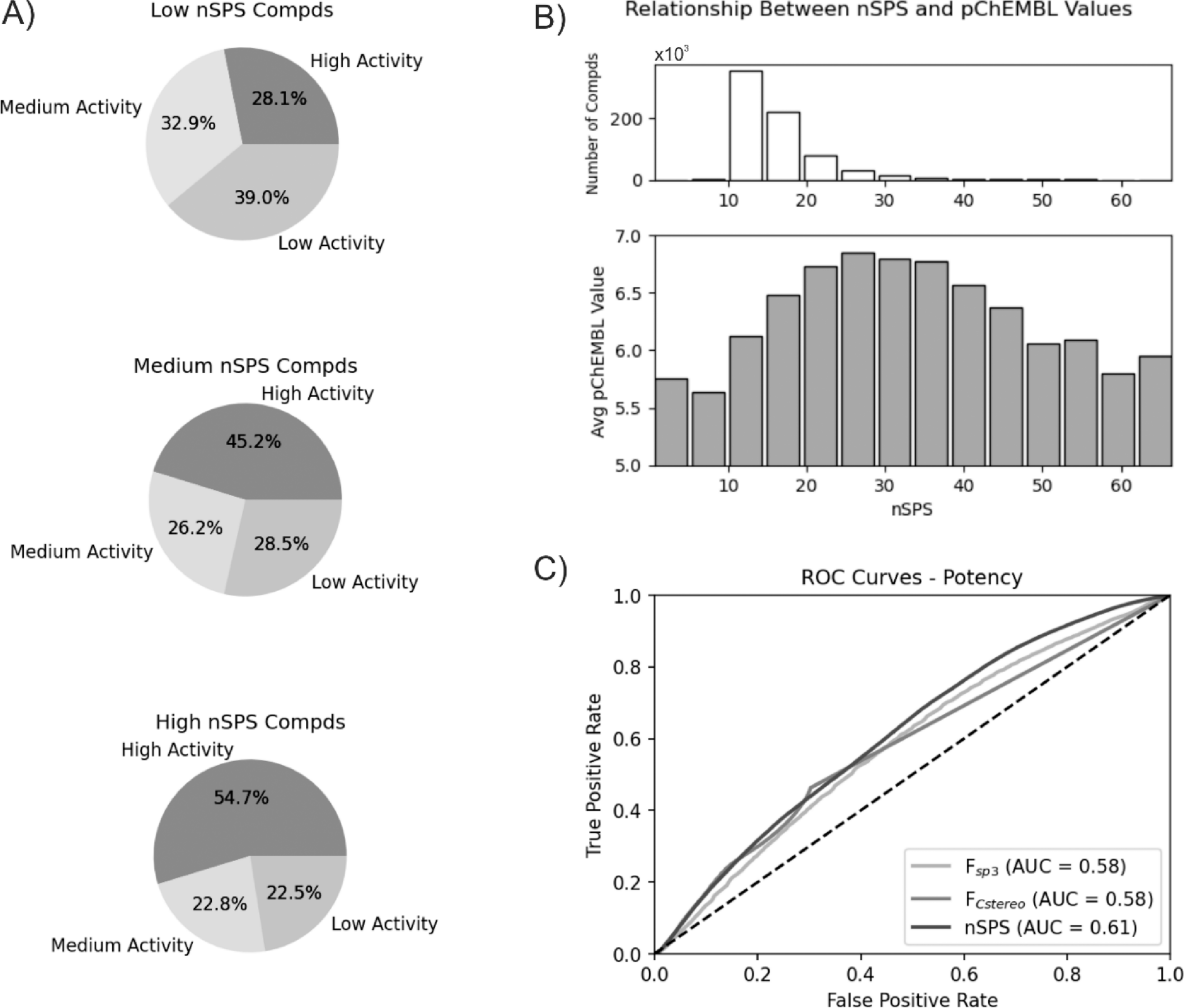
**A)** Proportions of high, medium, and low activity in ChEMBL assays for compounds at three ranges of nSPS. **B)** Relationship between nSPS and pChEMBL values, where compounds are grouped into bins according to their nSPS values. Average pChEMBL values are calculated for each bin, where each bin contains at least 10 compounds. The number of compounds in each bin in the top panel represents the number of molecules in the bins of the bottom panel. **C)** ROC plot for the ability of *F*_sp^3^_, *F*_Cstereo_, and nSPS systems to discriminate between compounds with high and low-to-moderate potency in ChEMBL assays. AUC = 0.5 indicates no discriminatory ability (dashed diagonal line).

The analysis revealed that molecules with medium or high *F*_sp^3^_ (above 0.3) tend to have higher activity in assays than compounds with high unsaturation (Figure S7A). We also observed that structures with medium values of *F*_Cstereo_ have the highest proportion of high compound activity (54%) compared to compounds with either no stereogenic carbons or compounds with high values of *F*_Cstereo_ (Figure S7B). The differences in activity between molecule bins with low, medium, and high nSPS values are highly conspicuous. Low nSPS is associated with a high proportion of low-activity compounds (39%) and a low proportion of highly active compounds (28%). The proportion of highly active structures increases for the molecules with medium nSPS (45%) and reaches a majority (55%) for structures with high nSPS. Compounds with high nSPS also have a low proportion of structures with low activity in assays (23%; Figure 3A). Further data analysis of the relationship between activity and nSPS revealed that, on average, the highest activity is achieved by compounds with nSPS values between ca. 20 and 40 (Figure 3B). A further increase in nSPS appears to be generally non-advantageous for compound potency and is analogous to the findings of Clemons et al.^6^ for highly complex molecules characterized by high *F*_Cstereo_. Thus, a molecular complexity in the nSPS range of 20–40 appears to be generally a “necessary but not sufficient” condition to obtain a ligand with an optimal assay activity. The average compound activity for different nSPS ranges has also been compared to the distribution of average molecular weights (Figure S8). Although generally low molecular weights correspond to low nSPS, the averaged weights appear to remain relatively constant (ca. 400–500) for compounds with nSPS above 10 units.

**Figure 4:**
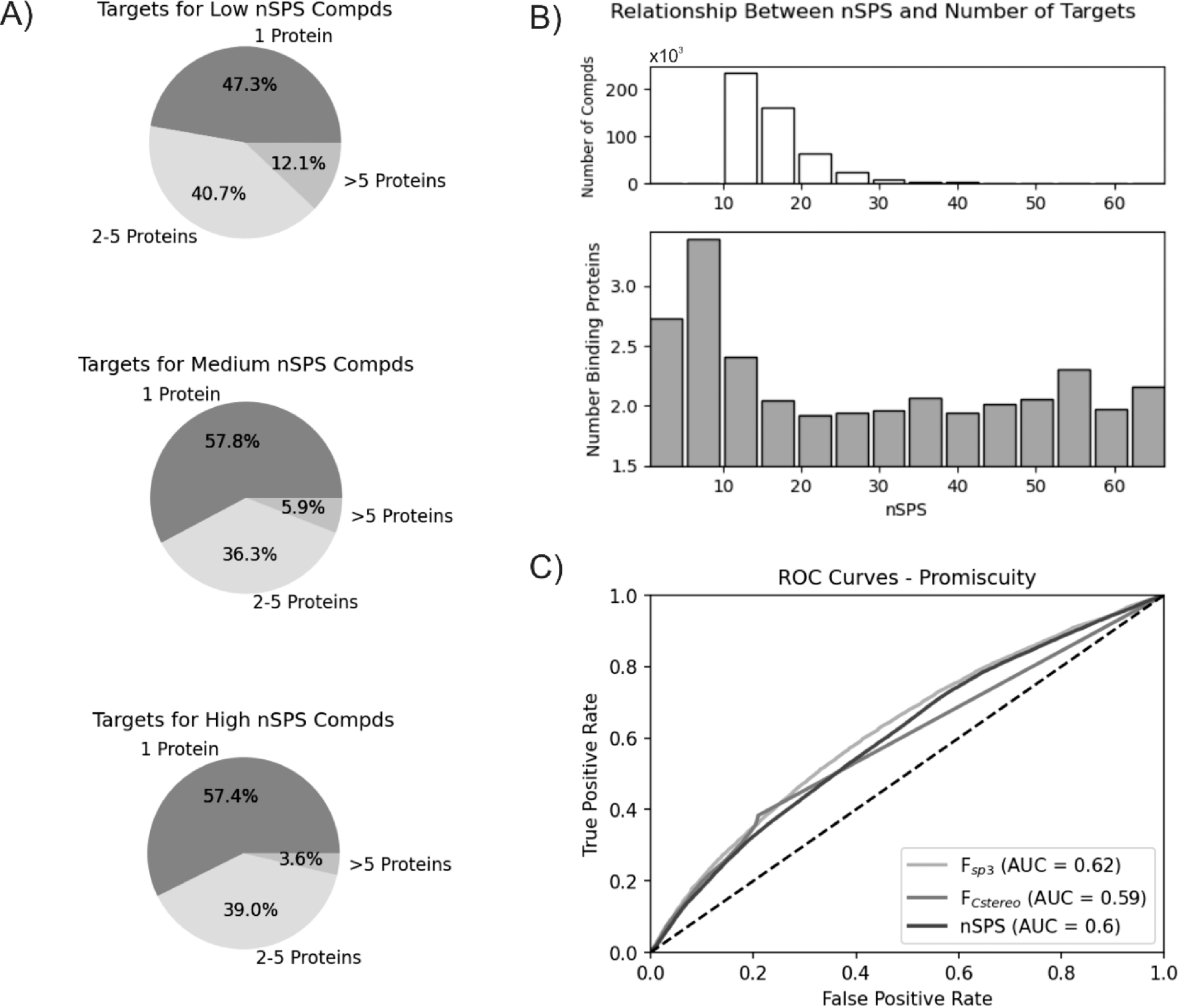
**A)** Proportions of number of targets for compounds at three ranges of nSPS, based on the ChEMBL data. **B)** Relationship between nSPS and the number of targets, where compounds are grouped into bins according to their nSPS values. The average number of bound proteins is calculated for each bin, where each bin contains at least 10 compounds. Numbers of compounds in each bin in the top panel represent the number of molecules in the bins of the bottom panel. **C)** ROC plot for the ability of *F*_sp^3^_, *F*_Cstereo_, and nSPS systems to discriminate between promiscuous and more target selective compounds. AUC = 0.5 indicates no discriminatory ability (dashed diagonal line).

A similar analysis was conducted for trends in target selectivity using compounds in the ChEMBL database. In order to render the analysis more reliable, the target selectivity analysis was performed only for compounds with the average pChEMBL > 5.3, thus excluding all weakly active compounds. By analogy to the classification used by Clemons et al.^6^ and Kombo et al.,^7^ the compounds were binned as highly selective (binding to only 1 protein), partially selective (binding 2–5 proteins), or promiscuous (binding 6 or more proteins). Compounds with higher values of *F*_sp^3^_ (>0.3) as well as *F*_Cstereo_ (>0.0) show a notable increase in the proportion of highly selective binders and a significant decrease in the proportion of promiscuous molecules (Figure S9). As in the case of the compound activity, the compounds with a moderate *F*_Cstereo_ range have a higher proportion of selective and lower proportion of promiscuous structures than compounds assigned a high *F*_Cstereo_ value. The obtained results for *F*_sp^3^_ and *F*_Cstereo_ are in agreement with previous reports.^6−8^ Structures with low nSPS have a relatively high proportion of promiscuity (12%) and a low proportion of highly selective compounds (47%). Molecules with medium nSPS have a decreased proportion of promiscuous compounds (6%) and increased proportion of compounds with only 1 binder (58%). Although the bin with the high nSPS values does not have a higher proportion of highly selective compounds, its fraction of promiscuous structures decreases to below 4% (Figure 4A). A closer investigation of the relationship between selectivity and nSPS indicated that there is a decreasing trend in the average number of binding proteins with an increase in the nSPS values up to 20. It thus seems that the most optimal nSPS values for target selectivity are 20 and above (Figure 4B).

The predictive powers of *F*_sp^3^_, *F*_Cstereo_, and nSPS were assessed with respect to compound potency and promiscuity using receiver operating characteristic curves (ROC) and calculated area under the curve (AUC). We tested how well these indicators perform in terms of binary classification into compounds with high and medium-to-low potency (Figure 3C), as well as into promiscuous and selective molecules (Figure 4C) in comparison to random choice (AUC = 0.5). nSPS gave the best AUC of 0.61 with respect to potency, whereas *F*_sp^3^_ and *F*_Cstereo_ each achieved AUC = 0.58. Similar performance was achieved for the classification with respect to promiscuity, where nSPS, *F*_sp^3^_, and *F*_Cstereo_ gave AUC = 0.60, 0.62, and 0.59, respectively. Although these results do not express high diagnostic ability, the positive performance of the assessed indicators is considerable and proves that nSPS has at least the same or better relation to potency and selectivity than the highly popular *F*_sp^3^_ and *F*_Cstereo_ metrics.

The analysis was continued by examining the average nSPS separately for experimental, investigational, and approved drugs in the 2023 structure sets provided by DrugBank.^35^ The experimental set contains compounds in the discovery and pre-discovery phases exhibiting drug-like properties, and the investigational data set comprises the drugs that reached clinical trials, while the approved drug set contains compounds that have been accepted for commercialization. The average nSPS values are similar and relatively high for all three sets. The lowest average nSPS of 18.2 was found for the experimental set. The investigational set has a slightly increased average nSPS of 19.3 units, whereas the approved drug set has the highest average nSPS of 20. We also performed analysis of the drugs approved by the FDA between 1951 and 2021, as summarized by Scott et al.^29^ Overall, the averages of *F*_sp^3^_, *F*_Cstereo_, and nSPS values do not appear to have changed appreciably over the years and show no apparent increasing or decreasing trends (Figure S10). The average nSPS of the approved drugs in this time period is relatively high and equals 21 units. The average nSPS values for the approved drugs seem to correspond well with the optimal nSPS range found for the compound potency and selectivity in the ChEMBL data set. Thus, for compounds intended for biological investigations and in the absence of any other indications, it appears that molecules with nSPS values in the range of 20–40 units will be particularly promising. As indicated by the statistical model of Hann et al.,^2^ and further discussed by Schuffenhauer et al.,^53^ there appears to be a trade-off between the probability of a random compound to have a measurable affinity to a target and the probability of finding a useful interaction with that target. Highly complex ligands may have a high affinity and selectivity for a target when a match is found, but the chance of a very complex and target-fitting molecule being present in a screening deck is relatively low. Hann et al. thus suggested that there is an optimal complexity range that allows us to obtain ligands complex enough to have a measurable binding affinity and good selectivity, but not too complex to have a very high likelihood of mismatch with the binding site. Thus, we suggest that, in the case of small libraries intended for screening of random scaffolds against a macromolecular target, the nSPS values should not be increased significantly over 20 units due to the increasing probability of a mismatch with the binding site for the higher scores. We recommend that the full, optimal nSPS range of 20–40 units is explored only in the case of high-throughput screening campaigns involving large numbers of compounds where the potential biological benefits of highly complex structures outweigh the difficulty in finding the hit.

Similarly to the Böttcher complexity index, the un-normalized SPS can also be applied for the assessment of spacial modifications occurring during generalized chemical transformations. The difference in SPS (ΔSPS) between the product and the starting material(s) for a particular reaction type can be easily calculated and used for comparison and ranking of contributions to spacial changes between different chemical reactions (Table 3).

**Table 3:**
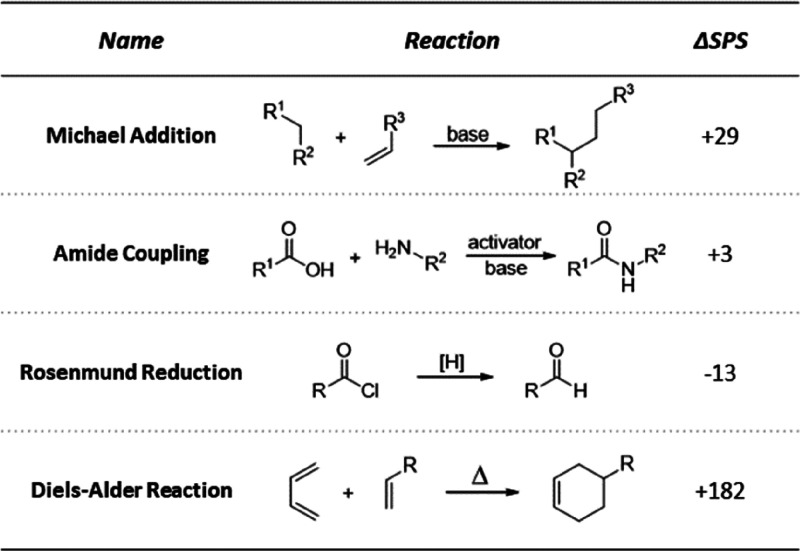
Changes in SPS between Products and Starting Materials for Exemplary Chemical Reactions (R Group ≠ H)

## Conclusions

Considering the high importance and widespread use of *F*_sp^3^_ and *F*_Cstereo_ indicators in medicinal chemistry and their link to biologically relevant compound features, we propose a new scoring system that not only combines the benefits of both *F*_sp^3^_ and *F*_Cstereo_ but also overcomes shortcomings caused by their simplistic nature. SPS and its size-normalized version nSPS provide a more granular evaluation for a diverse range of structures than *F*_sp^3^_ and *F*_Cstereo_, retaining strong correlation to both indicators but also explicitly accounting for such topological features as ring sizes and skeletal branching. Thus, the normalized spacial score appears to reflect more the chemist’s intuitive assessment of complexity regarding spacial arrangements in molecules. Furthermore, the nSPS is applicable in a biological context. Analysis of ChEMBL compounds showing activities in assays revealed trends of generally increasing selectivity and potency with increasing nSPS values. At very high nSPS values above 40, the impact on activity strength became unfavorable. The average nSPS of the FDA-approved drugs over the past seven decades was over 20 units and does not have a general rising or falling trend. Based on the observed results, in the absence of any known contradictions, we recommend to select for compounds with nSPS in the range of 20–40, which could potentially maximize the ligand potency and target selectivity, remembering that an increase in the average molecular complexity can have a negative impact on the assay hit rate. In addition, SPS can be used in the context of synthesis planning and analysis by directly comparing different chemical transformations and their impact on spacial arrangements and molecular complexity in the resulting products’ molecular structures.

## Experimental Section

### General Methods

The cheminformatics data analysis was performed using Python 3.8 and was based on RDKit software version 2021.09.3.^33^ Python script preparation and data analysis were done in Visual Studio Code version 1.64.2 (Microsoft Corporation, USA) using a virtual environment in conda, package, and environment manager, version 4.10.3. Data processing and analysis was performed with the help of NumPy (version 1.21.5), Pandas (version 1.3.5), and scikit-learn (version 1.0.1) packages. All presented plots were prepared with Matplotlib (version 3.5.1) and Seaborn (version 0.11.2). The Python script used to calculate SPS and nSPS based on SMILES has been made publicly available and can be accessed through the GitHub repository: https://github.com/frog2000/Spacial-Score. The Böttcher complexity scores were calculated using a modified Python script, originally developed by the Forli group,^26^ and modified by us to also account for the *E*/*Z* isomers, as described by Böttcher.^21^ The updated script, merged into the master branch, is available directly from the GitHub repository of the Forli group: https://github.com/forlilab/bottchscore. The Allu and Oprea complexity scores were calculated using a Python implementation by Voršilák and Svozil.^40^ The script is available from the GitHub repository: https://github.com/lich-uct/nonpher.

### Data Sets

Described data analysis was performed using several external data sets: experimental (6K compounds), investigational (4K compounds), and approved drugs (2K) from Drugbank, as well as combined experimental and approved drugs from DrugBank (8K compounds), Enamine advanced screening collection (527K compounds), NPs extracted from ChEMBL database version 29 (42K compounds), and compounds from the DCM set provided by Wassermann et al.^38^ (139K compounds), ChEMBL database version 30 (718K compounds after filtering), the GDB-17 compound set (randomly selected 100K compounds),^52^ and the FDA-approved compound set provided by Scott et al.^29^ (1112 entries).

The selection of NPs from ChEMBL 29 was conducted exactly as previously described by Grigalunas and colleagues.^54^

### Data Preparation

DrugBank, Enamine, DCM, and all ChEMBL compounds containing at least two heavy atoms were standardized by desalting the input structures, neutralizing charges, performing SMILES canonicalization, and deduplication. The script used for the standardization is available from https://github.com/mpimp-comas/2022_grigalunas_smo_anta (python_scripts). NPs from ChEMBL 29 were additionally deglycosylated as previously described by Grigalunas et al.^54^

The ChEMBL-based analysis concerning compound potency and selectivity was performed on molecules with an associated assay ‘confidence_score’ of 9. The compound filtering was performed using an appropriate SQL query with MySQL (version 8.0.27) relational database management system (see Supporting Information) from the full ChEMBL 30 locally installed database. The resulting data were loaded into the Pandas dataframe and merged with previously standardized SMILES for the compounds in the ChEMBL 30 database, joining the entries based on the ChEMBL ID for the compound. The entries which did not contain pChEMBL values or standardized SMILES were removed. All available pChEMBL values for the entries with the same target ID and the ChEMBL compound ID were averaged, allowing us to obtain a mean pChEMBL value across all performed assays for a specific compound interacting with a specific target. Duplicated entries for the same compound with the same target ID were then removed. The number of proteins that given a compound bound was based on the total number of entries with the same ChEMBL compound ID but different target IDs. The compounds in the Pandas dataframe were then deduplicated by the ChEMBL compound IDs, and this prepared dataframe was used for the analysis of a compound’s potency and target selectivity.

### Analysis of Compound Potency and Selectivity

The ChEMBL compounds were divided into three groups for each considered scoring system. For *F*_sp^3^_ the following bins were used: *F*_sp^3^_ ≤ 0.3, 0.3 < *F*_sp^3^_ < 0.6, and *F*_sp^3^_ ≥ 0.6. For *F*_Cstereo_: *F*_Cstereo_ = 0.0, 0.0 < *F*_Cstereo_ < 0.3, and *F*_Cstereo_ ≥ 0.3. For nSPS: nSPS ≤ 11.22 (25th percentile), 11.22 < nSPS < 17.68, and nSPS ≥ 17.68 (75th percentile). Compounds within each *F*_sp^3^_, *F*_Cstereo_, and nSPS class were further grouped as low (average pChEMBL ≤ 5.3), medium (5.3 < average pChEMBL < 6.5), or high potency (average pChEMBL ≥ 6.5), noting the percentage of each potency group within each complexity class. A similar process was followed for the investigation of compound selectivity, but here only molecules with average pChEMBL > 5.3 were considered. Compounds within each *F*_sp^3^_, *F*_Cstereo_, and nSPS class were further grouped as highly selective (1 known target), moderately selective (2–5 known targets), or promiscuous (6 or more known targets), also noting the percentage of each selectivity group within each complexity class.

Subsequently, the compounds were grouped based on their complexity index into bins where each bin had at least 10 members. Average pChEMBL values and average numbers of targets were calculated for each complexity bin, and then the average values were plotted against the complexity bins for nSPS. The number of compounds populating each bin was also recorded.

ROC curves and AUC values were generated using scikit-learn package, where the discriminatory abilities of nSPS, *F*_sp^3^_, and *F*_Cstereo_ to distinguish between either promiscuous/non-promiscuous compounds or high/medium-low compound potency were assessed.

Analogous analyses were performed based on size-normalized Böttcher complexity scores (see Supporting Information).

### Analysis of Correlations

Pearson correlations of SPS and nSPS with *F*_sp^3^_, *F*_Cstereo_, and molecular descriptors were calculated for a combined set of 12 000 unique compounds, where 3000 structures were sampled from each of the following data sets: DrugBank, Enamine, DCM, and NPs from ChEMBL. The PBF score was calculated for each molecule as an average for 10 representative 3D conformations generated using ETKDG version 3 as implemented in RDKit.^55^ NP-likeness and QED were calculated as previously described by Grigalunas et al.^54^ All remaining molecular descriptors were calculated based on the methods available from RDKit.

### Analysis of the FDA-Approved Compounds

FDA-approved compounds between 1951 and 2021 were assessed, calculating nSPS, *F*_sp^3^_, and *F*_Cstereo_ for each structure. Average complexity index values and standard deviations were calculated for molecules approved in each year.

## Supporting Information Available

Structures and complexity scores for selected organic molecules (S1, S4, S5); histogram of *F*_Cstereo_ scores for compounds in the reference sets (S2); 2D-histograms comparing distribution of nSPS with *F*_sp^3^_, *F*_Cstereo_ and the complexity score by Allu and Oprea (S3, S6); potency and selectivity results for compounds at different complexity levels (S7, S9, S12); relationship between nSPS, pChEMBL values and molecular weights (S8); changes of nSPS, *F*_sp^3^_ and *F*_Cstereo_ for FDA-approved drugs between 1951 and 2021 (S10); distributions of Böttcher complexity scores for reference compound sets (S11); ROC for potency and selectivity based on Böttcher complexity scores (S13); and SQL query for compound selection from ChEMBL30 database (PDF)

## Abbreviations Used

ASA: approximate surface area
AUC: area under the curve
*C*_m_: Böttcher complexity score
*C*_T_: Bertz total molecular complexity score
DCM: dark chemical matter
*F*_Cstereo_: fraction of stereogenic carbons
*F*_sp^3^_: fraction of sp^3^-hybridized carbons
HA: heavy atom
nSPS: normalized spacial score
NP: natural product
QED: quantitative estimate of drug-likeness
PBF: plane of best fit
ROC: receiver operating characteristic
*S*: Whitlock score
SPS: spacial score
