# Supplementary material for: Spacial Score—A Comprehensive Topological Indicator for Small-Molecule Complexity
Source: J Med Chem. 2023 Aug 31;66(18):12739–50. doi: 10.1021/acs.jmedchem.3c00689 (PMC10544027; doi:10.1021/acs.jmedchem.3c00689)
Supplement: Supplementary file 1 — jm3c00689_si_001.pdf [file jm3c00689_si_001.pdf]

Supporting Information

Spacial Score – A Comprehensive Topological Indicator for Small Molecule Complexity

Adrian Krzyzanowski,*[a, b, †] Axel Pahl,[c] Michael Grigalunas[a] and Herbert Waldmann*[a,b]

[a] Dr. A. Krzyzanowski, Dr. M. Grigalunas, Prof. Dr. H. Waldmann
Department of Chemical Biology
Max Planck Institute of Molecular Physiology
Otto-Hahn-Straße 11, 44227 Dortmund, Germany

[b] Dr. A. Krzyzanowski, Prof. Dr. H. Waldmann
Faculty of Chemistry, Chemical Biology
Technical University Dortmund
Otto-Hahn-Straße 6, 44221 Dortmund, Germany

[c] Dr. A. Pahl
Compound Management and Screening Center
Max Planck Institute of Molecular Physiology
Otto-Hahn-Straße 11, 44227 Dortmund, Germany

†Current Address:
GSK Medicines Research Centre,
Gunnels Wood Road, SG1 2NY Stevenage, U.K.

* Corresponding Authors:
H.W. Herbert.Waldmann@mpi-dortmund.mpg.de
A.K. adrian.x.krzyzanowski@gsk.com

Table of Contents

1. Analysis Results S3
2. SQL Query S12
3. Data Analysis Results with Böttcher Complexity Scores S13

1. Analysis Results

A)

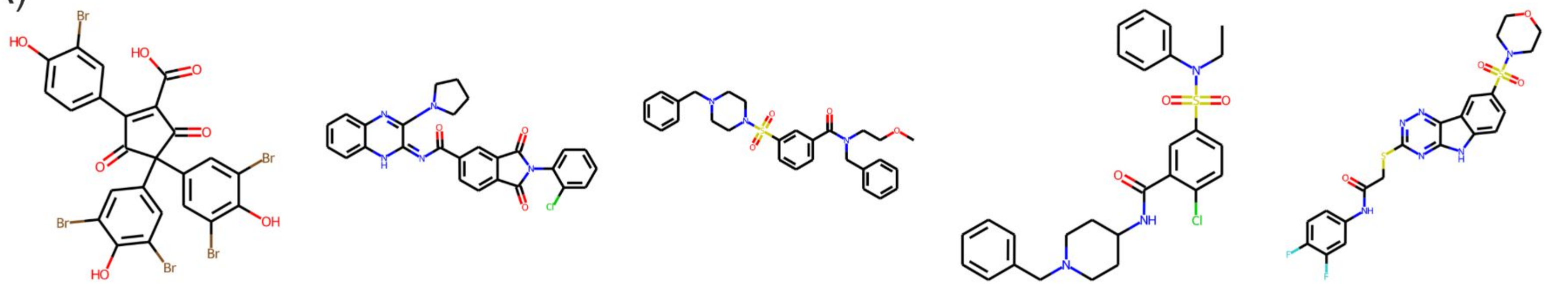

nSPS	15.03	nSPS	15.03	nSPS	15.03	nSPS	15.03	nSPS	15.03
SPS	541	SPS	541	SPS	541	SPS	526	SPS	526
Fcstereo	0.0	Fcstereo	0.0	Fcstereo	0.0	Fcstereo	0.0	Fcstereo	0.0
Fsp3	0.04	Fsp3	0.15	Fsp3	0.32	Fsp3	0.3	Fsp3	0.24

B)

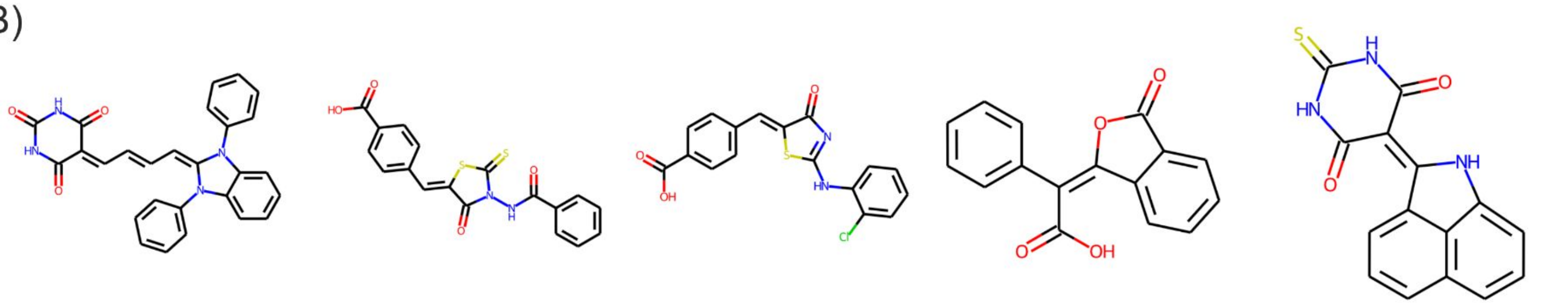

nSPS	15.35	nSPS	15.38	nSPS	15.46	nSPS	15.5	nSPS	15.62
SPS	522	SPS	400	SPS	371	SPS	310	SPS	328
Fcstereo	0.0	Fcstereo	0.0	Fcstereo	0.0	Fcstereo	0.0	Fcstereo	0.0
Fsp3	0.0	Fsp3	0.0	Fsp3	0.0	Fsp3	0.0	Fsp3	0.0

C)

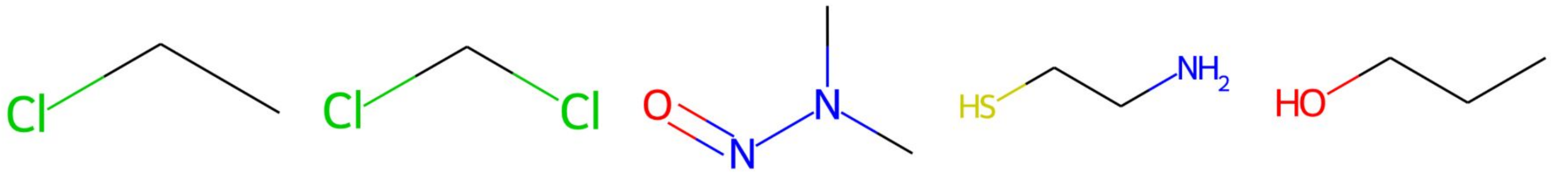

nSPS	6.0	nSPS	6.0	nSPS	6.8	nSPS	7.5	nSPS	7.5
SPS	18	SPS	18	SPS	34	SPS	30	SPS	30
Fcstereo	0.0	Fcstereo	0.0	Fcstereo	0.0	Fcstereo	0.0	Fcstereo	0.0
Fsp3	1.0	Fsp3	1.0	Fsp3	1.0	Fsp3	1.0	Fsp3	1.0

Figure S1. A) Examples of compounds with considerable topological complexities and $F_{Cstereo}$ equal zero. **B)** Examples of compounds with considerable topological complexities and both F_{sp3} and $F_{Cstereo}$ equal zero. **C)** Examples of simple compounds with the maximum possible F_{sp3} score of one.

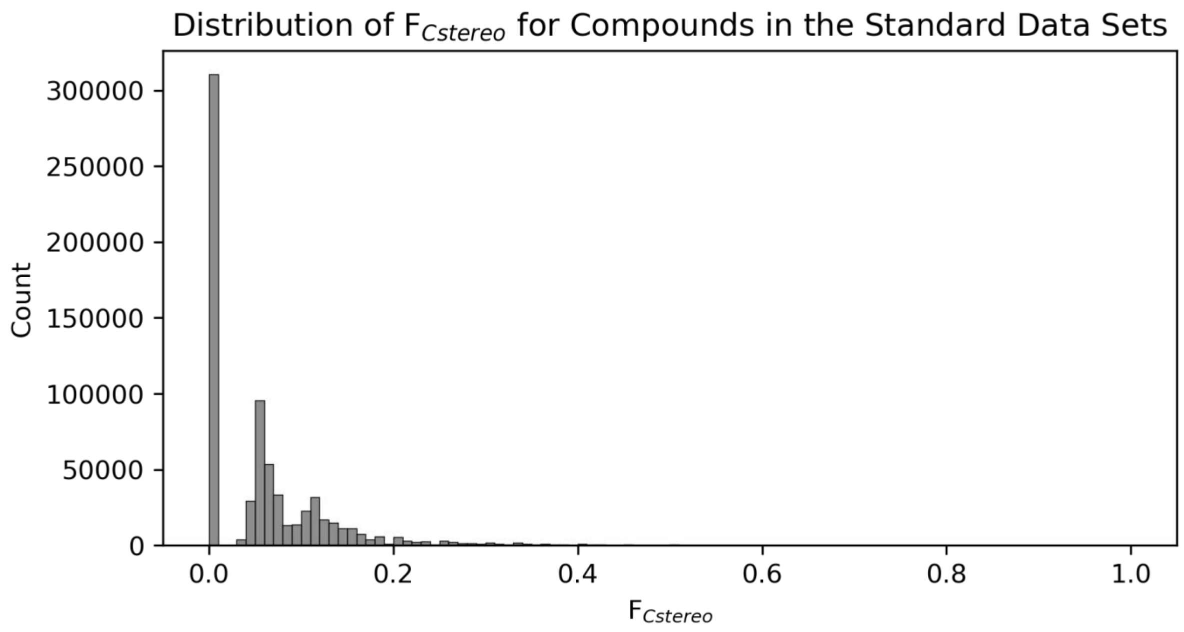

Figure S2. Histogram of $F_{Cstereo}$ scores for deduplicated combined compound collection from DrugBank, Enamine, Dark Chemical Matter and ChEMBL natural product data sets. 44% of all the considered molecules has $F_{Cstereo}$ score equal 0.0.

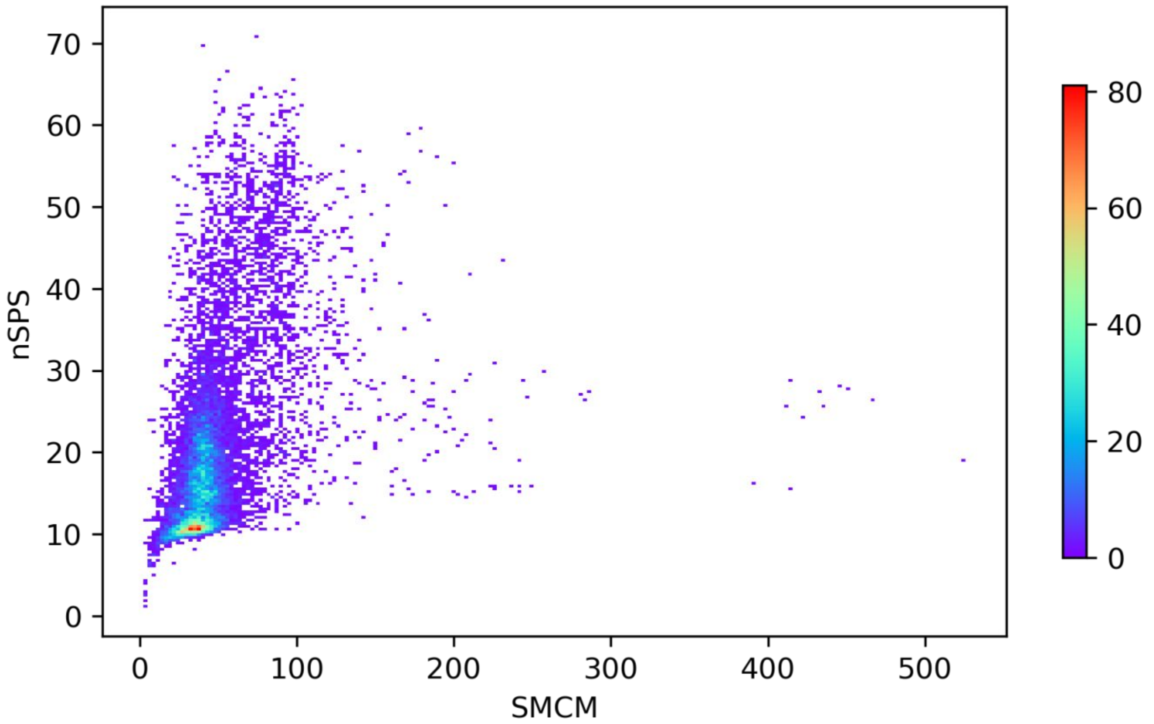

Figure S3. Relationship between nSPS and the scores for the Allu and Oprea index (SMCM). The SMCM scores were calculated based on the SMCM interpretation by Voršilák and Svozil. The plot is based on data for 12000 representative compounds selected in equal proportions from DrugBank, Enamine, Dark Chemical Matter and ChEMBL natural product data sets. The calculated Pearson correlation coefficient is equal to 0.46.

Within 1st nSPS percentile:

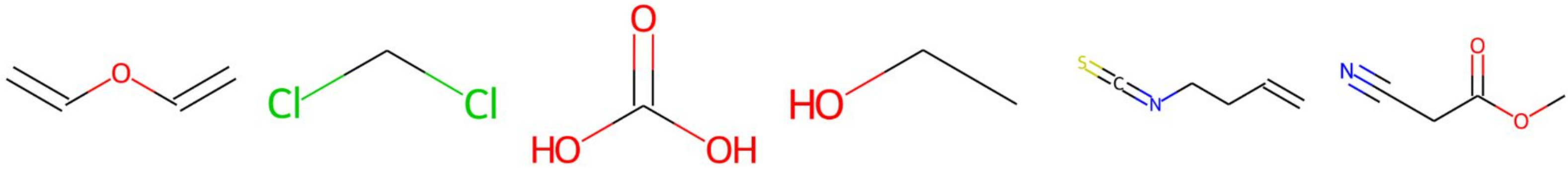

nSPS	5.6	nSPS	6.0	nSPS	6.0	nSPS	6.0	nSPS	6.86	nSPS	6.86
SPS	28	SPS	18	SPS	24	SPS	18	SPS	48	SPS	48
Fcstereo	0.0	Fcstereo	0.0	Fcstereo	0.0	Fcstereo	0.0	Fcstereo	0.0	Fcstereo	0.0
Fsp3	0.0	Fsp3	1.0	Fsp3	0.0	Fsp3	1.0	Fsp3	0.4	Fsp3	0.5

5th nSPS percentile:

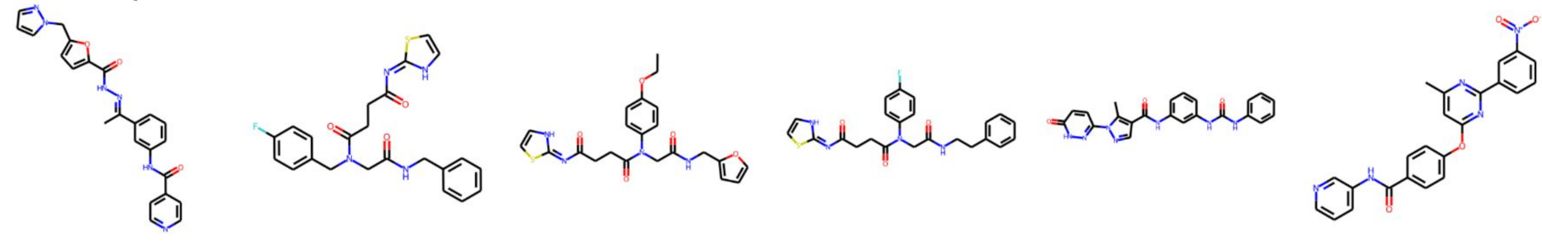

nSPS	10.41	nSPS	10.41	nSPS	10.41	nSPS	10.41	nSPS	10.41	nSPS	10.41
SPS	333	SPS	333	SPS	333	SPS	333	SPS	333	SPS	333
Fcstereo	0.0	Fcstereo	0.0	Fcstereo	0.0	Fcstereo	0.0	Fcstereo	0.0	Fcstereo	0.0
Fsp3	0.09	Fsp3	0.22	Fsp3	0.27	Fsp3	0.22	Fsp3	0.05	Fsp3	0.04

25th nSPS percentile:

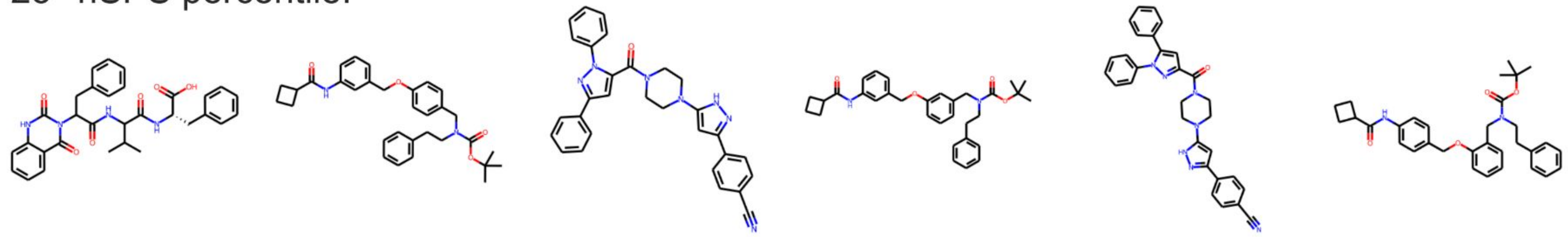

nSPS	13.34	nSPS	13.34	nSPS	13.34	nSPS	13.34	nSPS	13.34	nSPS	13.34
SPS	547	SPS	507	SPS	507	SPS	507	SPS	507	SPS	507
Fcstereo	0.1	Fcstereo	0.0	Fcstereo	0.0	Fcstereo	0.0	Fcstereo	0.0	Fcstereo	0.0
Fsp3	0.26	Fsp3	0.38	Fsp3	0.13	Fsp3	0.38	Fsp3	0.13	Fsp3	0.38

Figure S4. Examples of compounds with different values of nSPS: 0-1st, 5th and 25th percentile from DrugBank, Enamine, Dark Chemical Matter and ChEMBL natural product data sets.

50th nSPS percentile:

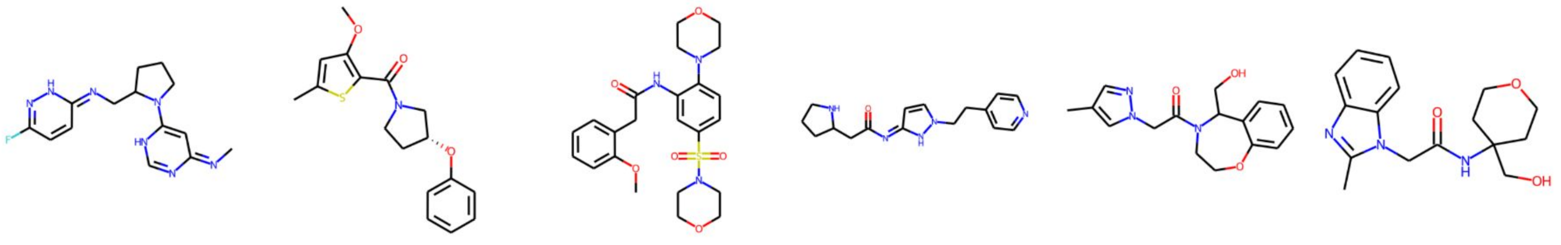

nSPS	17.55	nSPS	17.55	nSPS	17.55	nSPS	17.55	nSPS	17.55	nSPS	17.55
SPS	386	SPS	386	SPS	579	SPS	386	SPS	386	SPS	386
Fcstereo	0.07	Fcstereo	0.06	Fcstereo	0.0	Fcstereo	0.06	Fcstereo	0.06	Fcstereo	0.0
Fsp3	0.43	Fsp3	0.35	Fsp3	0.43	Fsp3	0.44	Fsp3	0.38	Fsp3	0.5

75th nSPS percentile:

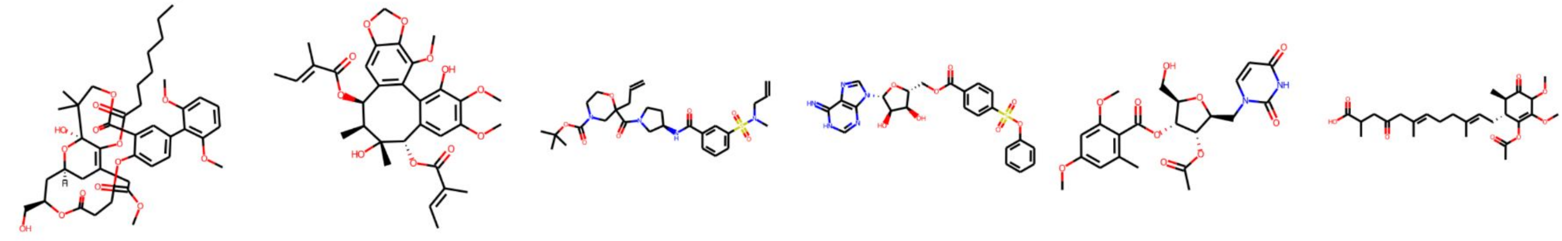

nSPS	21.67	nSPS	21.67	nSPS	21.68	nSPS	21.68	nSPS	21.68	nSPS	21.68
SPS	1192	SPS	932	SPS	867	SPS	802	SPS	737	SPS	737
Fcstereo	0.07	Fcstereo	0.12	Fcstereo	0.07	Fcstereo	0.17	Fcstereo	0.18	Fcstereo	0.15
Fsp3	0.56	Fsp3	0.44	Fsp3	0.54	Fsp3	0.22	Fsp3	0.45	Fsp3	0.62

99th nSPS percentile:

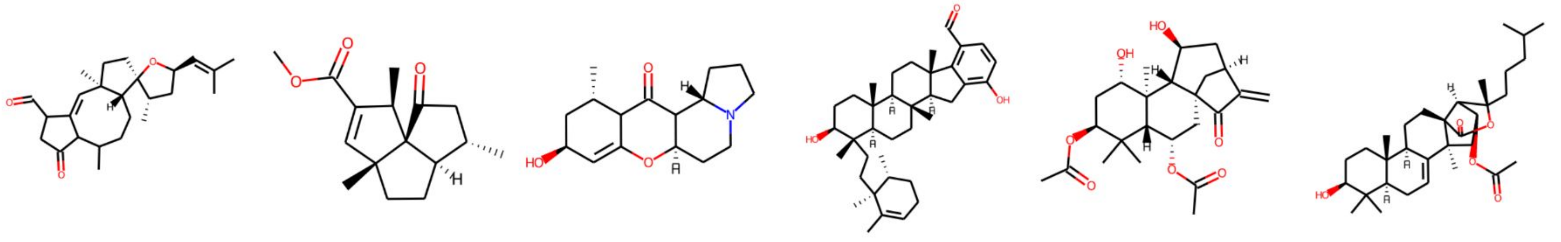

nSPS	47.89	nSPS	47.89	nSPS	47.9	nSPS	47.9	nSPS	47.9	nSPS	47.89
SPS	1341	SPS	910	SPS	958	SPS	1916	SPS	1485	SPS	1772
Fcstereo	0.32	Fcstereo	0.31	Fcstereo	0.38	Fcstereo	0.27	Fcstereo	0.38	Fcstereo	0.28
Fsp3	0.76	Fsp3	0.75	Fsp3	0.81	Fsp3	0.76	Fsp3	0.79	Fsp3	0.88

Figure S5. Examples of compounds with different values of nSPS: 50th, 75th and 99th percentile from DrugBank, Enamine, Dark Chemical Matter and ChEMBL natural product data sets.

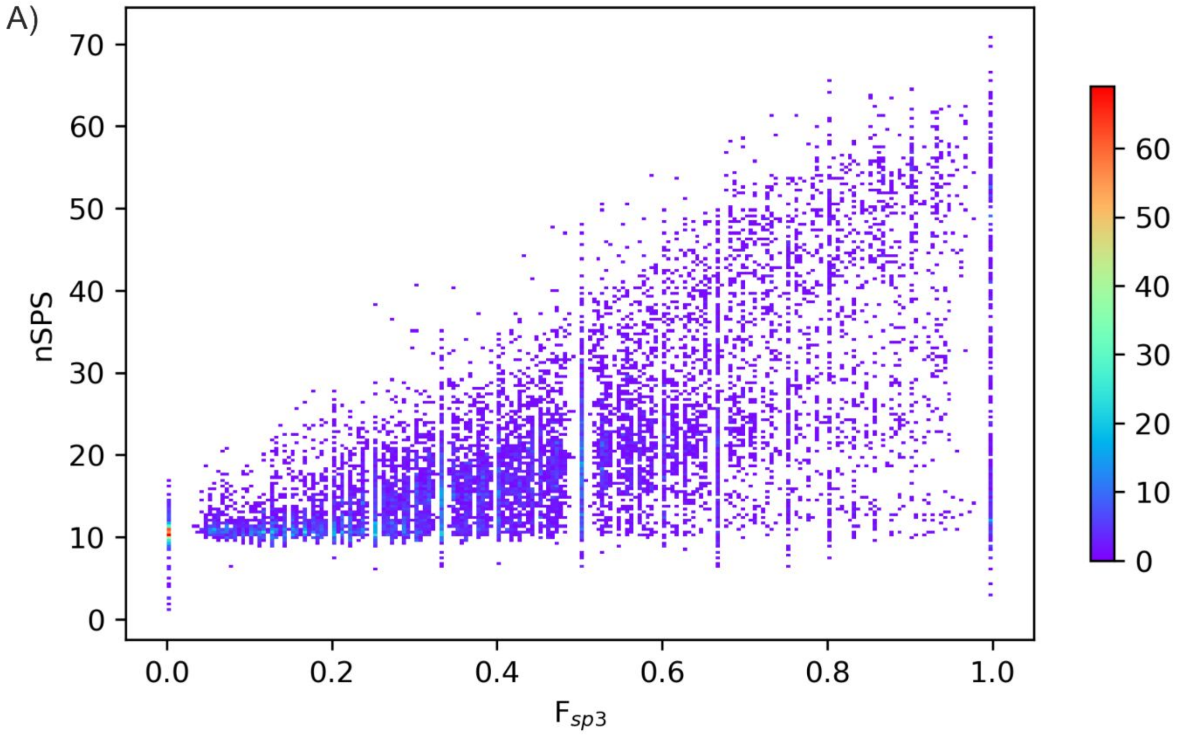

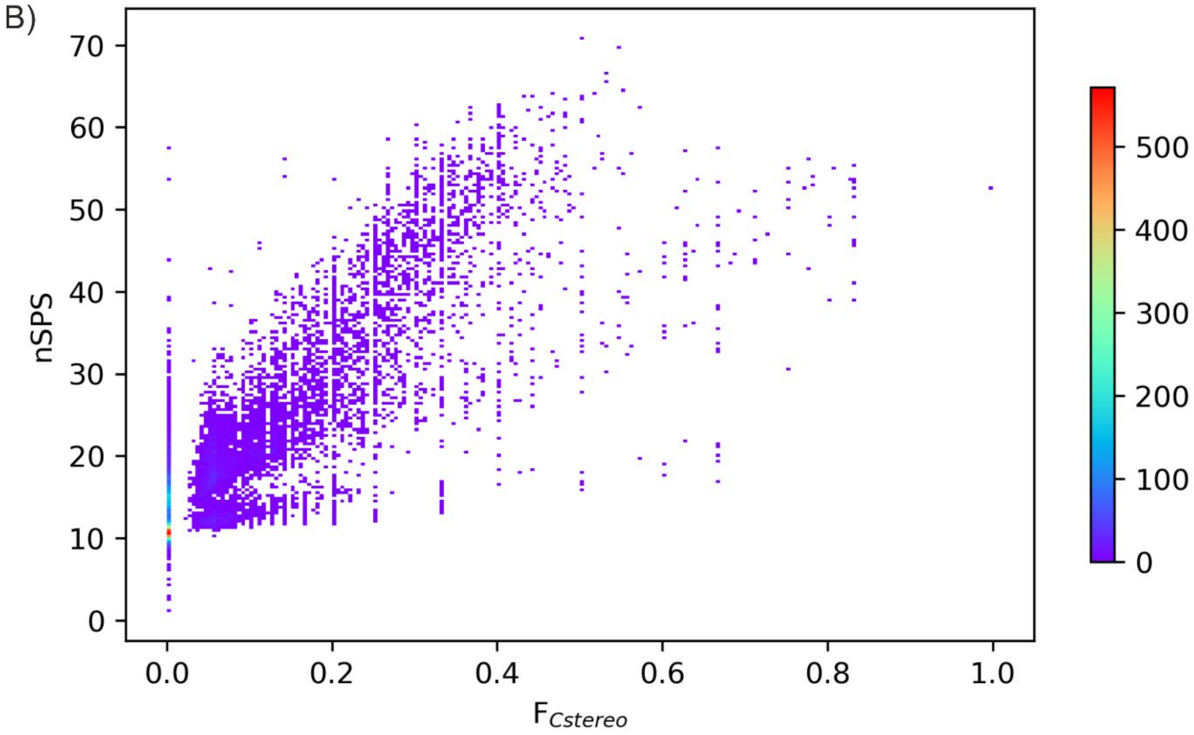

Figure S6. A) Relationship between nSPS and F_{sp3}. The plot is based on data for 12000 representative compounds selected in equal proportions from DrugBank, Enamine, Dark Chemical Matter and ChEMBL natural product data sets. **B)** Relationship between nSPS and $F_{Cstereo}$. The plot is based on data for 12000 representative compounds selected in equal proportions from DrugBank, Enamine, Dark Chemical Matter and ChEMBL natural product data sets.

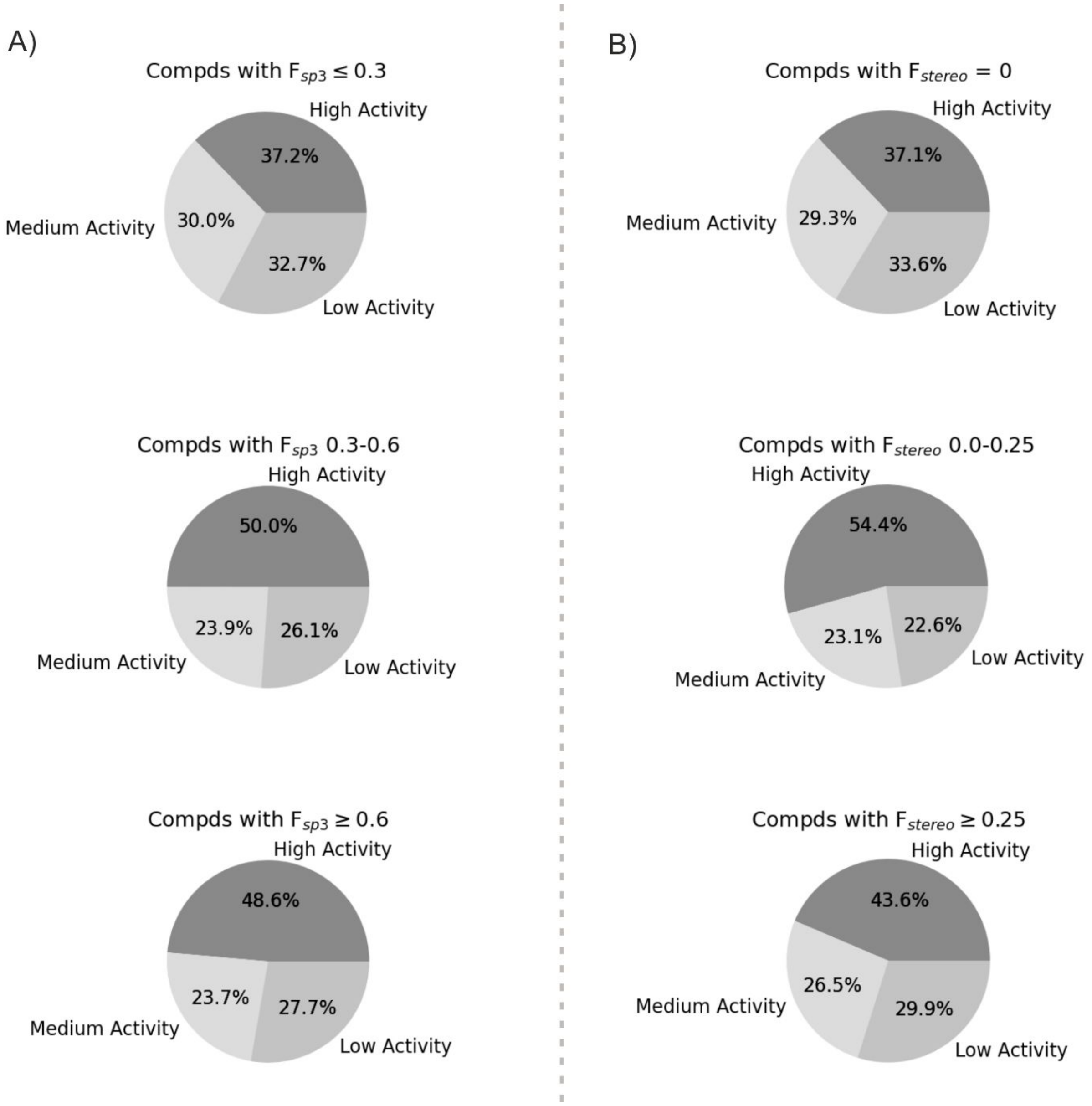

Figure S7. A) Proportions of high, medium and low activity in ChEMBL assays for compounds at three ranges of F_{sp3} scores. **B)** Proportions of high, medium and low activity in ChEMBL assays for compounds at three ranges of $F_{Cstereo}$ scores.

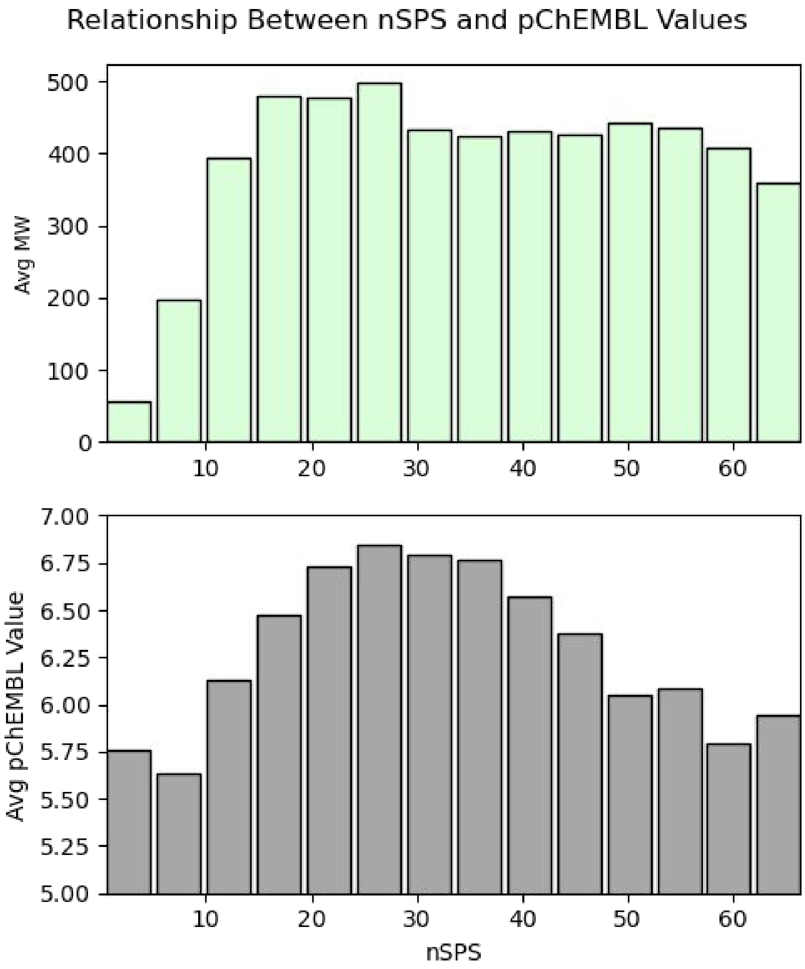

Figure S8. Relationship between nSPS and pChEMBL values and nSPS and molecular weight, where compounds are grouped into bins according to the their nSPS values. Average pChEMBL and molecular weight values are calculated for each bin, where each bin contains at least ten compounds. The bins in the top panel corresponds the bins of the bottom panel, and they represent the same molecules.

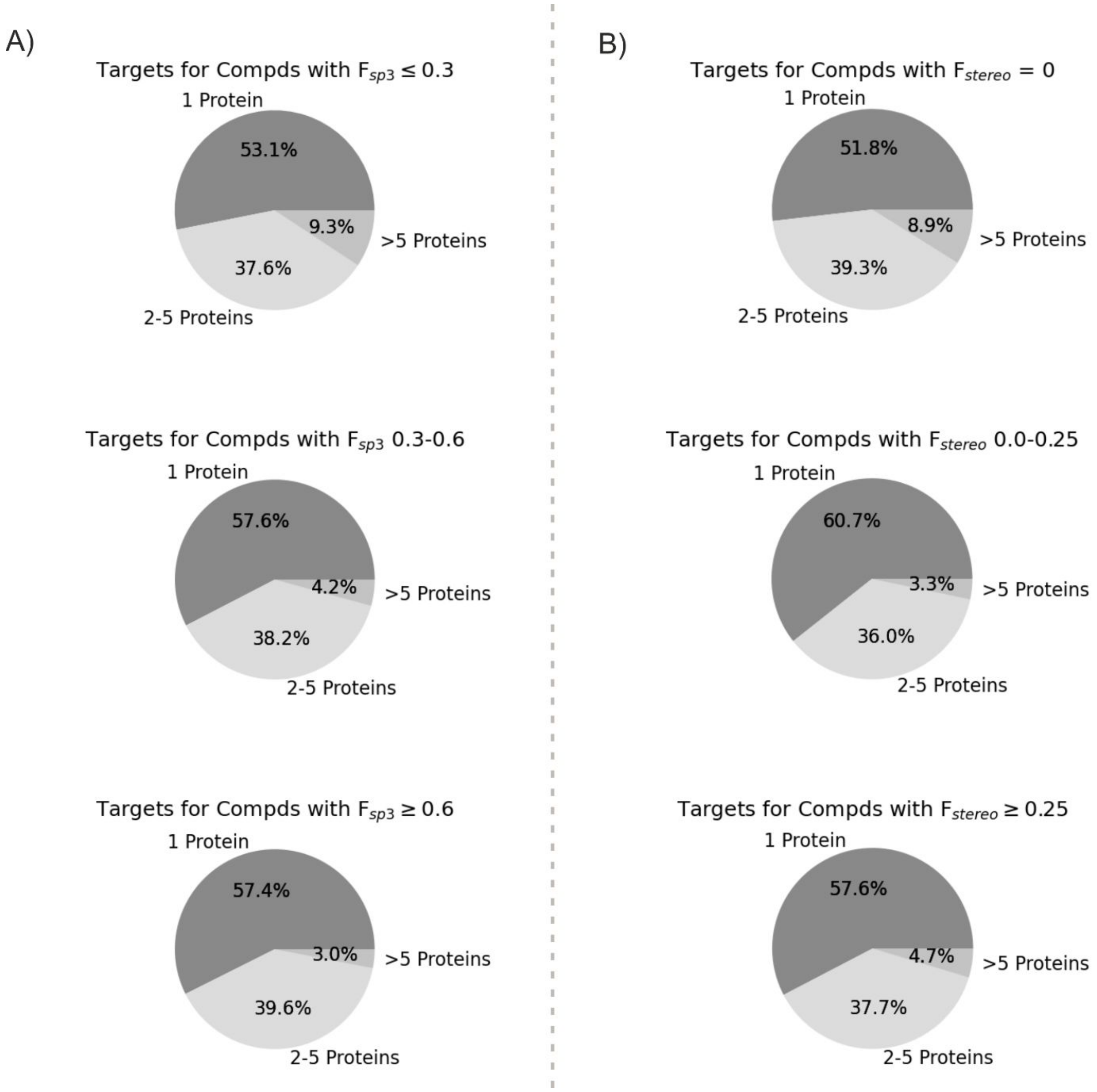

Figure S9. A) Proportions of number of targets for compounds at three ranges of F_{sp3} scores, based on the ChEMBL data. **B)** Proportions of number of targets for compounds at three ranges of $F_{Cstereo}$ scores, based on the ChEMBL data.

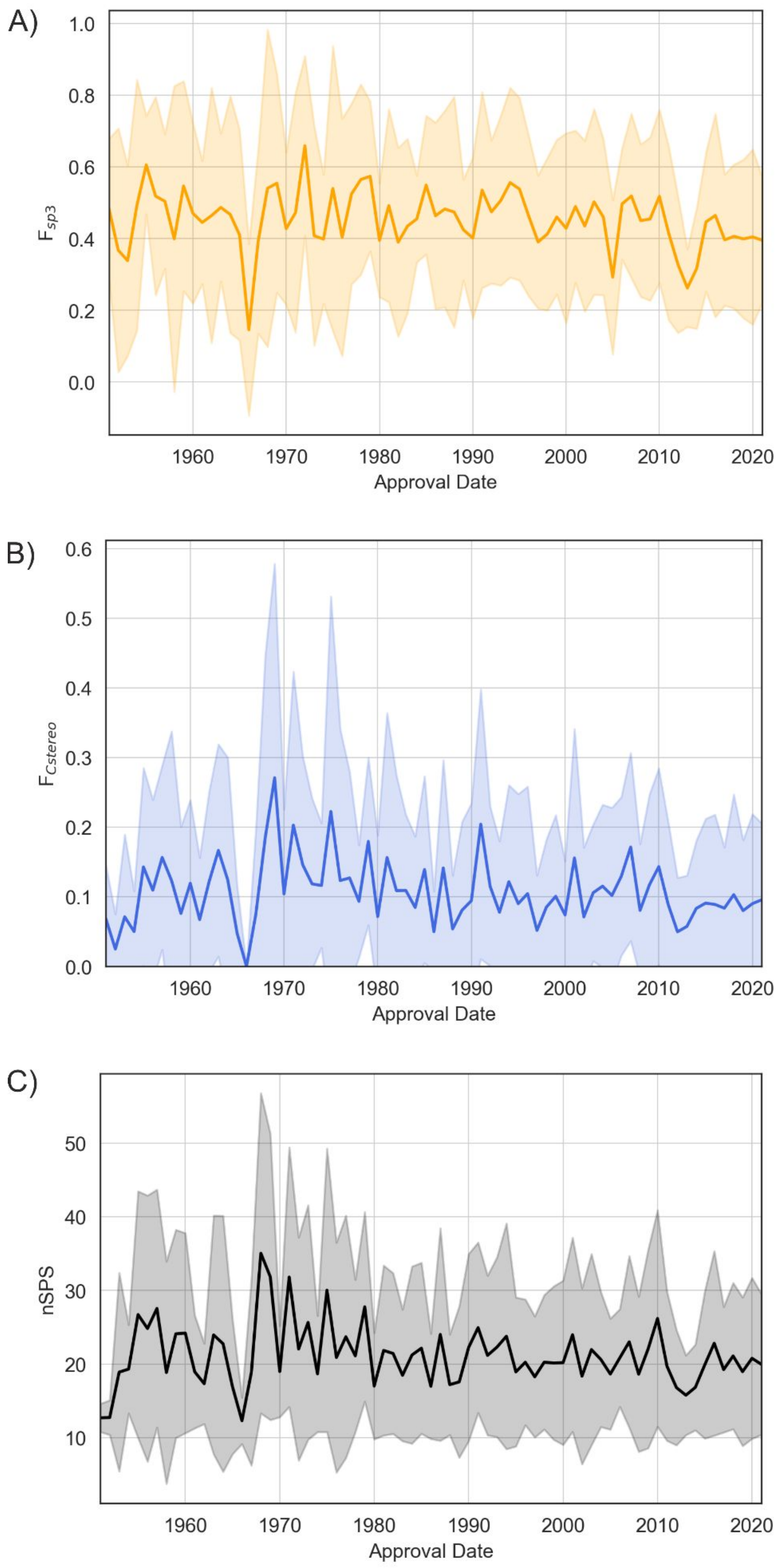

Figure S10. A) Average F_{sp3} for FDA-approved drugs over time. Shaded area shows ±1 standard deviation. **B)** Average $F_{Cstereo}$ for FDA-approved drugs over time. Shaded area shows ±1 standard deviation. **C)** Average nSPS for FDA-approved drugs over time. Shaded area shows ±1 standard deviation.

2. SQL Query

The SQL query (MySQL dialect) used to extract information from ChEMBL database (version 30) about compounds with the associated assay confidence score of 9:

```
SELECT md.chembl_id, cs.canonical_smiles, cs.standard_inchi, cs.standard_inchi_key,
a.tid, a.confidence_score, act.pchembl_value, td.pref_name, pfc.protein_class_desc,
pfc.l1, pfc.l2, pfc.l3, pfc.l4, pc.pref_name as pref_name2, pc.short_name

FROM activities act LEFT JOIN compound_records cr on act.record_id = cr.record_id
LEFT JOIN molecule_dictionary md on act.molregno = md.molregno
LEFT JOIN compound_structures cs on md.molregno = cs.molregno
LEFT JOIN assays a on act.assay_id = a.assay_id
LEFT JOIN target_dictionary td on a.tid = td.tid
LEFT JOIN target_components tc on td.tid = tc.tid
LEFT JOIN component_class cc on tc.component_id = cc.component_id
LEFT JOIN protein_classification pc on cc.protein_class_id = pc.protein_class_id
LEFT JOIN protein_family_classification pfc on cc.protein_class_id =
pfc.protein_class_id
WHERE a.confidence_score = 9;
```

3. Data Analysis Results with Böttcher Complexity Scores

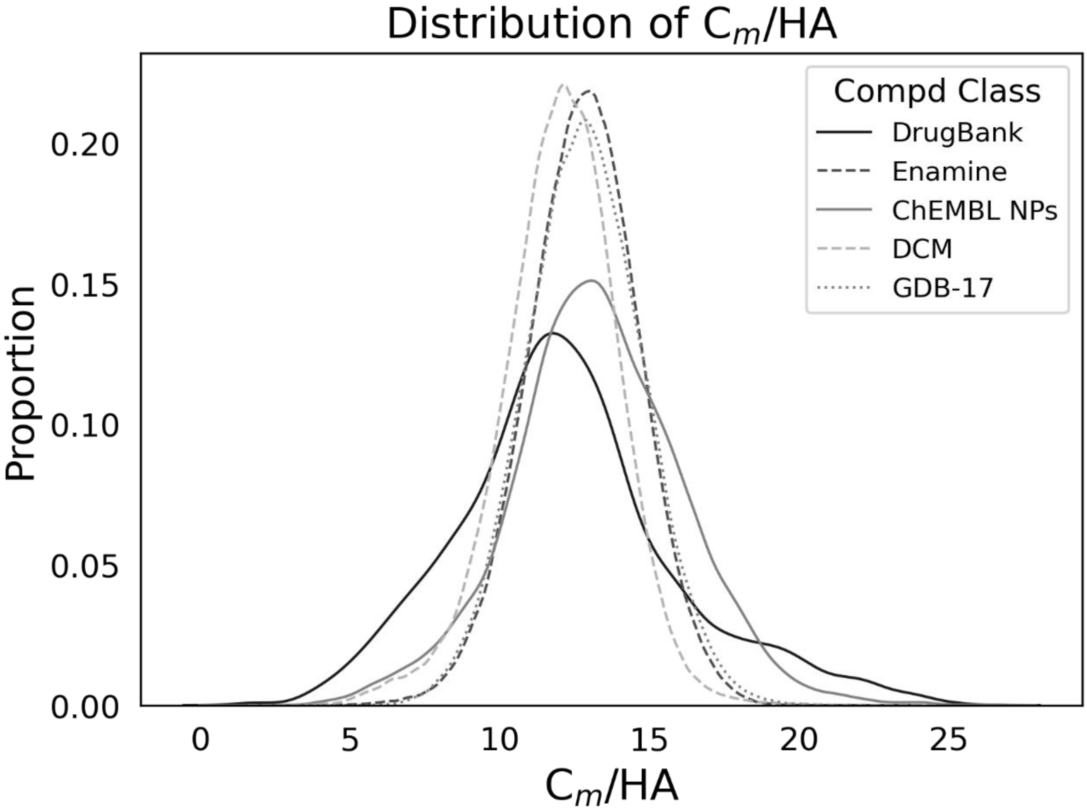

Figure S11. Comparison of distributions of Böttcher complexity scores (C_m) normalised by the number of heavy atoms (HA) for different data sets. The normalisation is applied to account for the size differences between the molecules in the data sets. There is little differentiation between the distributions.

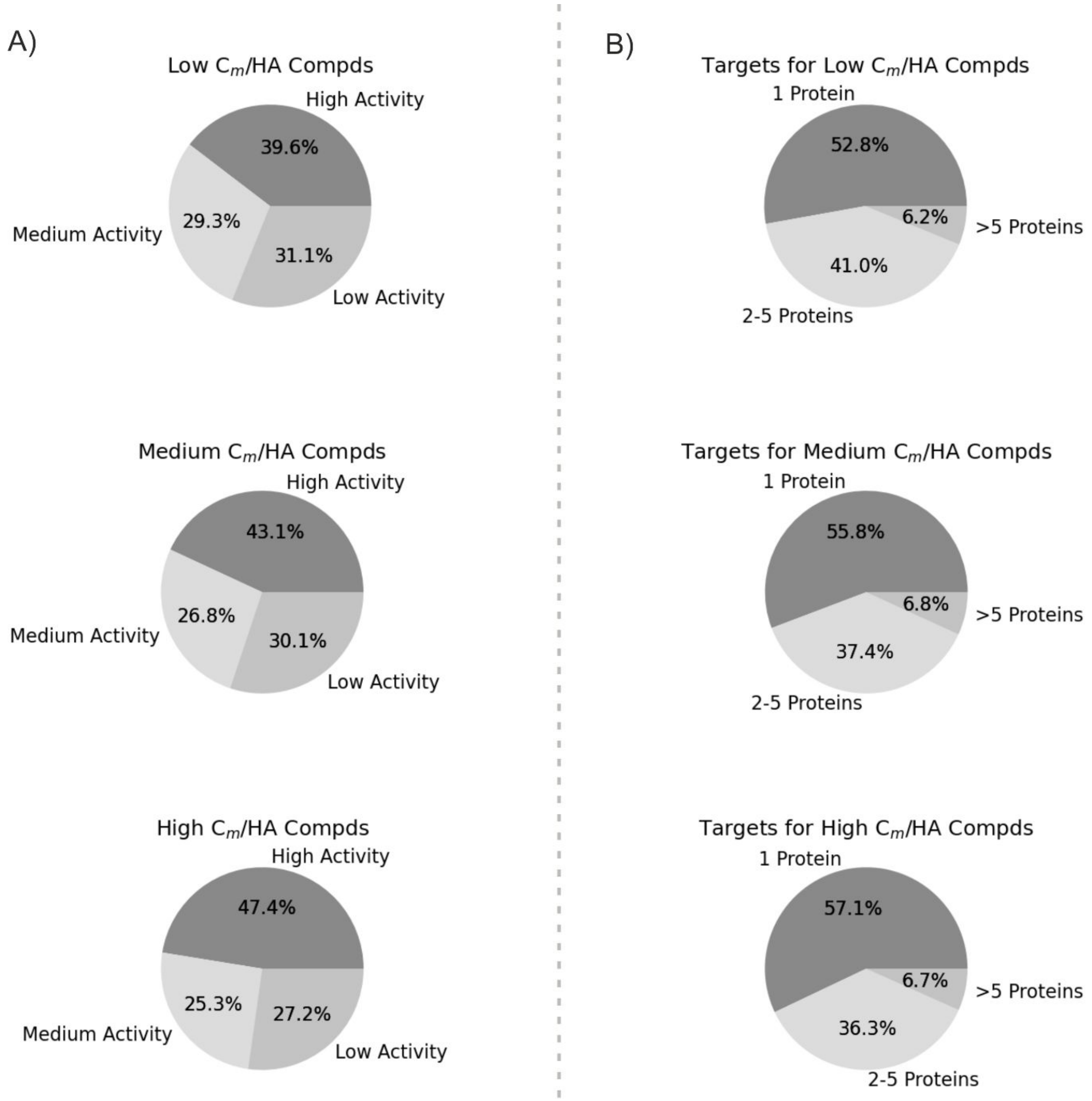

Figure S12. A) Proportions of high, medium and low activity in ChEMBL assays for compounds at three ranges of size-normalised Böttcher complexity scores (C_m/HA). **B)** Proportions of number of targets for compounds at three ranges of C_m/HA scores, based on the ChEMBL data. The following classification criteria were applied: low compound complexity (25% percentile): $C_m/HA \leq 10.84$; medium complexity: $10.84 < C_m/HA < 13.35$; high complexity (75th percentile): $C_m/HA \geq 13.35$.

A)

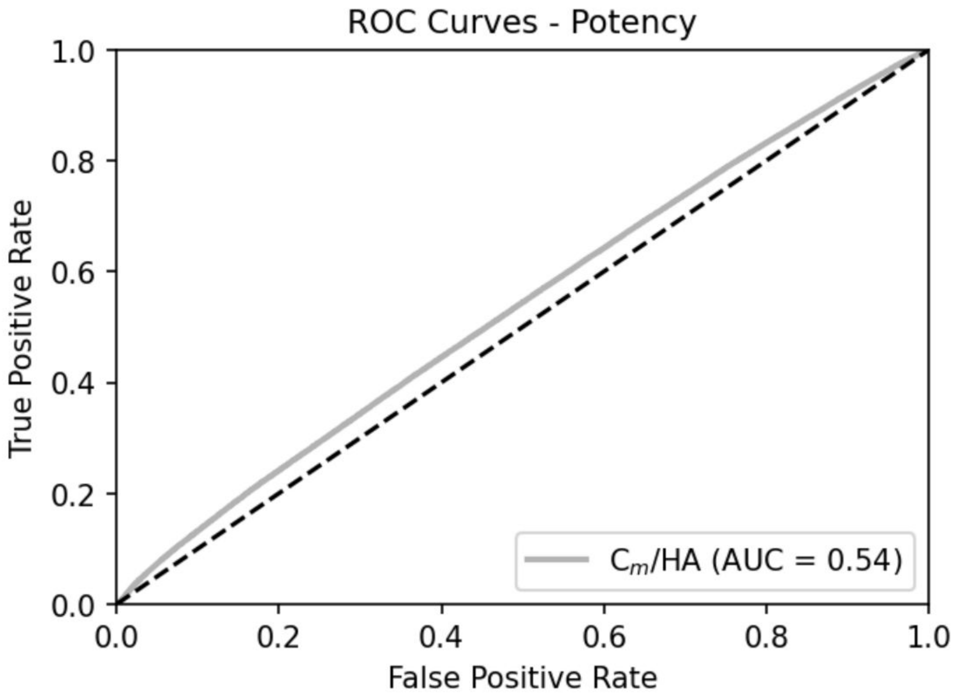

B)

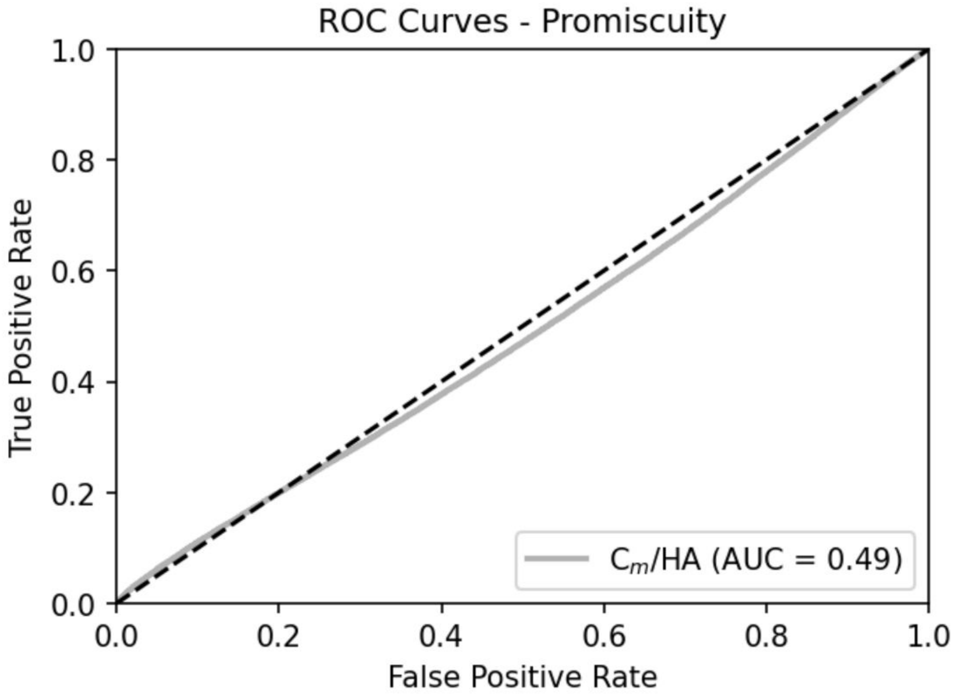

Figure S13. A) ROC plot for the ability of the size-normalised Böttcher complexity scores (C_m/HA) to discriminate between compounds with high and low-to-moderate potency in ChEMBL assays. AUC of 0.5 indicates no discriminatory ability (dashed diagonal line). Based on the analysed data, the size-normalised Böttcher complexity score has little or no application as a classifier in respect to compound potency. **B)** ROC plot for the ability of C_m/HA to discriminate between promiscuous (6 or more known binding targets) and more target selective compounds (1-5 binding targets). AUC of 0.5 indicates no discriminatory ability (dashed diagonal line). Based on the analysed data, the size-normalised Böttcher complexity score has no application as a classifier in respect to compound selectivity.
